# Expanding the Toolbox of Simple, Cost-Efficient, and Automatable Methods for Quantifying Surface Functional Groups on Nanoparticles—Potentiometric Titration

**DOI:** 10.1021/acsmeasuresciau.5c00062

**Published:** 2025-08-20

**Authors:** Isabella Tavernaro, Philipp C. Sander, Elina Andresen, Uwe Schedler, Ute Resch-Genger

**Affiliations:** † 42220 Federal Institute for Materials Research and Testing (BAM), Division Biophotonics, Richard-Willstaetter-Str. 11, 12489 Berlin, Germany; ‡ PolyAn, Schkopauer Ring 6, 12681 Berlin, Germany

**Keywords:** potentiometric and optical pH titration, cost-efficient screening methods, quantification of surface amino groups, amorphous silica nanoparticles, quantitative nuclear magnetic resonance spectroscopy, optical fluorescamine assay, automation

## Abstract

Measuring surface functional groups (FGs) on nanomaterials (NMs) is essential for designing dispersible and stable NMs with tailored and predictable functionality. FG screening and quantification also plays a critical role for subsequent processing steps, NM long-term stability, quality control of NM production, and risk assessment studies and enables the implementation of sustainable and safe­(r)-by-design concepts. This calls for simple and cost-efficient methods for broadly utilized FGs that can be ideally automated to speed up FG screening, monitoring, and quantification. To expand our NM surface analysis toolbox, focusing on simple methods and broadly available, cost-efficient instrumentation, we explored a NM-adapted pH titration method with potentiometric and optical readout for measuring the total number of (de)­protonable FGs on representatively chosen commercial and custom-made aminated silica nanoparticles (SiO_2_ NPs). The accuracy and robustness of our stepwise optimized workflows was assessed by several operators in two laboratories and method validation was done by cross-comparison with two analytical methods relying on different signal generation principles. This included traceable, chemo-selective quantitative nuclear magnetic resonance spectroscopy (qNMR) and thermogravimetric analysis (TGA), providing the amounts of amino silanes released by particle dissolution and the total mass of the surface coatings. A comparison of the potentiometric titration results with the reporter-specific amounts of surface amino FGs determined with the previously automated fluorescamine (Fluram) assay highlights the importance of determining both quantities for surface-functionalized NMs. In the future, combined NM surface analysis with optical assays and pH titration will simplify quality control of NM production processes and stability studies and can yield large data sets for NM grouping that facilitates further developments in regulation and standardization.

## Graphical abstract

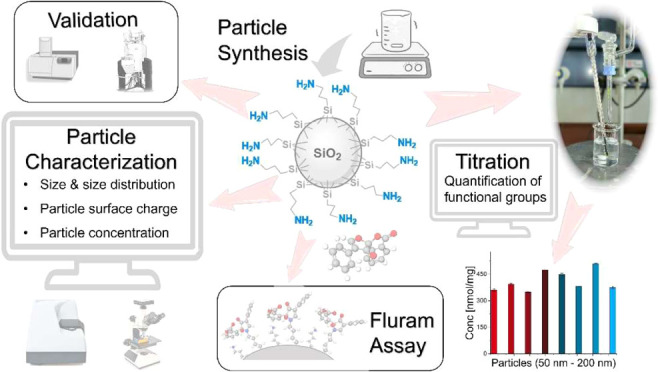

## 1 Introduction

Engineered nanomaterials (NMs) with varying chemical composition, size, morphology, crystallinity, and surface chemistry have reached a multibillion dollar market with applications in, e.g., sensing, medical diagnostics, catalysis, optoelectronics, energy conversion and storage, as well as in many consumer products.^1,2^ Most of these intrinsic physicochemical properties are accessible with meanwhile broadly available and increasingly standardized methods of different complexity and information content,^1,3,4^ and routinely reported for custom-made and commercial NMs. These properties are also needed for establishing relationships of NM properties in risk assessment and performance studies.^5–7^ Despite the long-standing focus on nanomaterial (NM) composition and size, the importance of NM surface chemistry has been underestimated. However, surface chemistry is crucial for determining dispersibility, stability, reactivity, processability, and interactions with biological and environmental systems. For a significant time, surface chemistry and its influence was only little considered due to its complexity and analytical challenges although it plays a central role in NM functionality and safety.^6^ The extensive research on surface modification strategies for functional NMs such as luminescent nanocrystals like semiconductor quantum dots and lanthanide based NMs, optically active noble metal NMs, and magnetic metal oxide NMs, where surface properties are closely linked to material performance and NM application, triggered a broad interest in NM surface analysis in the last years.^8–13^ This also initiated the search for analytical methods, which enable the reliable quantification of functional groups (FGs) and ligands on different types of NMs.^6,14–16^ Versatile, yet advanced methods for surface analysis are X-ray photoelectron spectroscopy (XPS) and mass spectrometry (MS) methods, such as time-of-flight MS, that both measure dried NMs deposited on solid supports *in vacuo*, with a high sensitivity, but a limited potential for quantification in the case of the latter.^17–19^ Lately, chemo-selective nuclear magnetic resonance spectroscopy (NMR), providing detailed bulk and quantitative information, has emerged as method for characterizing coordinatively bound ligand shells on very small, diamagnetic semiconductor quantum dots in solution^20–22^ or rarely on gold NMs.^23–25^ Also surface ligands of other surface functionalized diamagnetic NMs such as silica nanoparticles (SiO_2_ NPs) have been meanwhile determined with quantitative solution NMR (qNMR), requiring NM removal from dispersion, followed by NM drying, weighting, and dissolution steps for ligand and FG quantification.^26–29^ All these sophisticated methods for surface analysis need cost-intensive instrumentation and well-trained operators. Other methods for surface analysis are thermogravimetric analysis (TGA), semiquantitative Raman and infrared (IR) spectroscopy, and zeta potential measurements.^30–33^ The latter simpler method, which yields information on surface charge and colloidal stability, is commonly performed during the initial NM characterization, and often employed to qualitatively monitor surface functionalization reactions. However, there exists no direct correlation between the zeta potential and the number of charged FGs.^34^

For the regular monitoring and process control of NM syntheses as well as for stability and aging studies, methods are needed, that provide fast quantitative information on surface chemistry with simple, broadly availability, and cost-efficient instrumentation, and can be ideally automated with affordable commercial tools. This need has been expressed by NM producers, particularly by small and medium sized enterprises (SMEs), as well as associations such as the NanoMesureFrance Association and the nanosafety community. This recently encouraged us to examine the potential of the exemplary chosen optical fluorescamine (Fluram) assay for quantifying surface FGs on different types of aminated NMs and demonstrate its automation with commercial cost-efficient instrumentation,^35^ with the goal to establish a validated tool for homogeneity and stability studies of custom-made surface functionalized nanomaterials. Optical assays, that can be performed with different types of dyes such as simple dyes bearing orthogonal reactive groups for the covalent attachment to surface FGs, chromogenic or fluorogenic dyes or cleavable reporters and read out with different optical instrumentation such as spectrometers, microplate readers or for sufficiently large particles even flow cytometers,^6,36^ yield only the number of FGs that are accessible for interaction and reaction with the selected signal-generating dye reporter. The measured amount is influenced by the reporter’s size, shape, and charge, and therefore represents only an assay- and reporter-specific measurand.^6^ In addition, particle properties such as size and morphology can affect reporter-FG interactions in a material-specific manner. Also, the optical properties of the particle, such as size- and refractive-index-dependent scattering, absorption, and fluorescence, can interfere with assay readout, particularly for reporters that are measured while bound to larger scattering particles or to colored and/or fluorescent particles.^6,28,37–41^ Nevertheless, the frequently measured amount of reporter accessible surface FGs is relevant for all applications involving subsequent NM labeling and allows to compare different NM batches and monitor NM stability. However, it does not necessarily allow a direct comparison with the results obtained with other analytical methods targeting different measurands.^38,39,42^

As an expansion of our NM surface chemistry toolbox of simple and cost efficient screening methods, we explored an NM-adapted pH titration method for measuring the total number of (de)­protonable FGs on aminated SiO_2_ NPs, frequently utilized in the life and material sciences.^43–45^ Such fast and nondestructive titration methods, which exploit ultrasmall protons or hydroxyl ions as reporters, can be adapted to other FGs, such as carboxylic acids, carbonyls, and thiols.^6,43,46^ In the following, we present the development and stepwise optimization of this simple back-titration method and demonstrate its applicability for the exemplarily chosen screening of surface amino FGs on custom-made and commercial aminated SiO_2_ NPs of different size and amino FG density. Its reliability and robustness were demonstrated by independent measurements of several operators in two laboratories. Method validation was performed by cross-comparison with solution qNMR and TGA, measuring chemo-selectively the total amount of surface amino FGs and nonselectively the thermally induced total mass loss of the organic surface coating. Subsequent comparison with the amount of reporter-accessible surface amino FGs obtained by our automated Fluram assay highlights the importance to measure both application-relevant NM surface properties. Overall, our results demonstrate the advantages of simple multimethod characterization workflows for the screening, monitoring, and quantification of surface FGs on NMs for fast and application-focused NM characterization, which can be simply automated with broadly accessible instrumentation. Our results are expected to pave the road for facilitating and accelerating NM surface analysis, and to support the reliable generation of large data sets for NM grouping. This will aid sustainable and safe­(r)-by-design approaches, as well as NM performance and risk assessment studies.

## 2 Experimental Section

### 2.1 Materials and Methods

All chemicals, reagents, and solvents were of analytical grade or higher and used as received, unless otherwise specified, while all aqueous solutions and buffers were prepared with ultrapure water (Milli-Q-water, 0.055 μS·m^–1^; Merck Milli-Q IQ 700 device). Commercial nonporous aminated SiO_2_ NPs were purchased from NanoComposix (USA, NC-50 (lot number: LBE0060)), NC-80 (lot number: SAM0152), NC-100 (lot number: MPP0063), NC-120 (lot number: JEA0209), Kisker Biotech GmbH & Co. KG (Germany, PSi-0.1 (lot number:GK2381443-01)), micromod Particle Technology GmbH (Germany, Sicastar50 (lot number: 1311443-01), Sicastar70 (lot number: 2851343-01), Sicastar100 (lot number: 2841343-01), Sicastar200 (lot number: 2361343-01)), microparticles GmbH (Germany, micro450, lot number: SiO_2_-NH_2_ AR114-2) or the NanoChOp project (NChOp-01 and NChOp-06),^47^ as ethanolic or aqueous dispersions.

### 2.2 SiO_2_ Nanoparticle Synthesis and Surface Modification

The custom-made SiO_2_ NPs (BAM SiO_2_-100 NH_2_ high) were synthesized by a sol–gel process previously described,^48^ and functionalized with (3-aminopropyl)­triethoxysilane (APTES, abcr GmbH, Germany) in ethanol (EtOH, Labsolute, Th Geyer, Germany) under Ar atmosphere and ambient conditions, using a postsynthetic grafting step.^35^ In addition, Klebosol particles (30R50) from Merck KGaA (Germany), plain SiO_2_ NPs (PSi-0.08, lot number: GK2881343-01) from Kisker Biotech GmbH & Co. KG, and amorphous, plain SiO_2_ NPs from PlasmaChem GmbH (Germany) were custom-modified with an amino silane (Klebosol-NH_2_, PSi-0.8 NH_2_ (1–6), SiO_2_ NH_2_-50, SiO_2_ NH_2_-100).

### 2.3 Nanoparticle Characterization

Particle size, colloidal stability, and surface charge of the custom-made and commercial SiO_2_ NPs were characterized by dynamic light scattering (DLS) and zeta potential measurements using a Malvern Panalytical Zetasizer Nano ZS equipped with a 633 nm laser. Aqueous dispersions of the SiO_2_ NPs were also characterized by nanoparticle tracking analysis (NTA) using the NanoSight LM 10 system (Malvern Panalytical, Germany) equipped with a 405 nm laser to determine the number-based hydrodynamic diameter (*d*_h,0_) and the particle number concentrations (PNC). All measurements were performed in triplicate at a temperature of 25 °C. The PNC was validated gravimetrically by measuring the mass fraction of SiO_2_ NPs in one vial of each batch in triplicate by drying 0.25 mL of the NP dispersion in plastic centrifuge tubes (2 mL, safe lock Eppendorf tubes, Eppendorf GmbH, Germany) overnight at 100 °C. The specific surface area of the SiO_2_ NPs and the theoretical monolayer was estimated, using a silica density of 2.20 g/cm^3^ for the particles obtained by NanoComposix and 2.0 g/cm^3^ for all the other particles.

### 2.4 Quantification of the Reporter-accessible and Total Number of Amino FGs

The reporter-accessible number of surface amino FGs was determined with an optical assay, recently semiautomated by us,^35^ using the dye precursor 4′-phenylspiro­[2-benzofuran-3,2′-furan]-1,3′-dione (fluorescamine, Fluram) which selectively reacts with primary amino groups. For the determination of the total amount of (de)­protonable surface amino FGs by pH titration, detailed in the Supporting Information (SI), the aminated SiO_2_ NPs were removed from aqueous or ethanolic dispersion by centrifugation (Hettich Rotina 380R, Germany) with 15,000 rcf for 30 min, followed by drying, weighing, and incubation in 0.001 M HCl for 1–3 h at room temperature (r.t.). After particle removal by centrifugation (15,000 rcf, 30 min), the supernatant was titrated with 0.001 M NaOH. The equivalence point was determined potentiometrically with a very sensitive pH electrode (InLab Micro Pro-ISM, Mettler Toledo, Germany) and a calibrated pH meter (Seven Excellence S475 pH meter, Mettler Toledo, Germany) or visually using an indicator dye (phenolphthalein or bromothymol blue, Sigma-Aldrich, Germany). The amount of 0.001 M NaOH solution required to reach the equivalence point of the acid–base-titration was utilized to calculate the amount of surface amino FGs on the aminated SiO_2_ NP. Data evaluation was done with a custom-made software. All measurements were typically conducted in triplicate at a constant temperature of 23 ± 1 °C. The results of the pH titrations were validated by comparison with solution qNMR and TGA. For the qNMR measurements, performed on a 600 MHz JEOL ECZ spectrometer, the centrifuged particles were dried overnight at 80 °C, weighed, and dissolved in 1 M sodium deuteroxide solution (NaOD, Sigma-Aldrich, Germany) in D_2_O (Sigma-Aldrich, Germany) at 50 °C for 4 h, before a known amount of maleic acid (TraceCERT, Sigma-Aldrich, Germany) was added as internal standard (2H, 6.3 ppm).^28^ TGA measurements were carried out with a calibrated NEXTA Series Simultaneous Thermogravimetric Analyzer (STA200RV, Hitachi, Japan) equipped with an autosampler, using a measurement procedure from 30 to 1000 °C.

The particle synthesis and characterization, as well as the quantification of amino FGs are detailed in the SI.

## 3 Results & Discussion

Measuring application- and safety-relevant NM properties is essential for process control, quality assurance, reproducibility, and for studies on long-term stability, exposure, and toxicity. As an extension of our screening platform for NM surface analysis and our work on the automation of optical assays for FG quantification,^35^ we explored different methods for the screening and determination of the total number of surface FGs. Thereby, we aimed for cost-efficient, and versatile techniques, suited for a comparison with established or increasingly utilized label-free methods for surface analysis such as XPS, qNMR, and TGA.^6,28,37^ One of the simplest method for obtaining the number of these (de)­protonable groups presents a pH titration, which can be monitored either electrochemically (potentiometric titration) or visually with an indicator dye.^43,44^ Both methods utilize very inexpensive, broadly available instrumentation, such as a glass buret, standardized titrants, and optionally a colored indicator or electrode for pH measurements. Due to very small sizes of the protons and hydroxyl ion reporters, pH titrations are expected to provide the total amount of (de)­protonable surface FGs. The assumption of a complete protonation (or deprotonation) of all surface FGs was previously confirmed by us for a series of carboxylated poly­(methyl methacrylate) (PMMA) nano- (NPs) and microparticles (MPs) varying in surface carboxyl FG amount and morphology with a conductometric method, which was validated by comparison with solid state NMR.^39^ This electroanalytical method, which measures solely sample conductivity, is, however, not suited for surface modified metal oxide NMs like silica as it cannot distinguish hydroxy, silanol, and carboxyl or amino FGs contrary to the titration method explored in this study.

An overview of our simple and automatable multimethod concept for surface FG screening and quantification assessed for aminated SiO_2_ NPs of varying size and surface amino FG amount, and its validation by method cross-comparison with qNMR and TGA is shown in Figure 1. Prior to surface analysis, the exemplarily studied sets of aminated SiO_2_ NPs shown in Figure 1a, sourced from commercial suppliers (NC, Sicastar, PSi-0.1 NH_2_, micro-450), the European research project NanoChOp (NChOp-01; NChOp-06),^47^ and custom-made batches (SiO_2_ NH_2_-50; Klebosol-NH_2_; PSi-0.8 NH_2_(1–6), BAM SiO_2_-100 NH_2_ high; SiO_2_ NH_2_-100), were first characterized by DLS, zeta potential, and NTA measurements (Figure 1b). These fast and nondestructive NM characterization methods, widely used in research and industry, require minimum sample preparation, and are well suited for real-time measurements as well as online, in-line, and at-line monitoring in automated workflows. The information on particle size, surface charge, and particle number concentration (PNC), detailed in the SI in Figures S1–S7, is also required for determining the total particle surface area. This enables the calculation of an estimated monolayer of surface amino FGs,^26^ assuming 4 APTES molecules per nm^2^ as broadly used in the literature as a theoretical upper limit for our calculation of a monolayer coverage.^49–51^ This theoretical monolayer coverage on hydroxylated silica surfaces which is calculated based on molecular footprint estimations under ideal conditions, e.g., a flat surface, complete hydrolysis, and absence of steric hindrances. Please note that naturally experimental values can vary for different particle types due to variations in surface curvature, hydroxyl group density, and reaction conditions. This assumption of 4 APTES molecules per nm^2^ in turn allows for distinguishing between aminated SiO_2_ NPs with mono- and multilayer amino silane surface shells. The results of the particle characterization and the estimated monolayers for the aminated SiO_2_ NPs are summarized in Tables S1–S3 in the SI.

**Figure 1:**
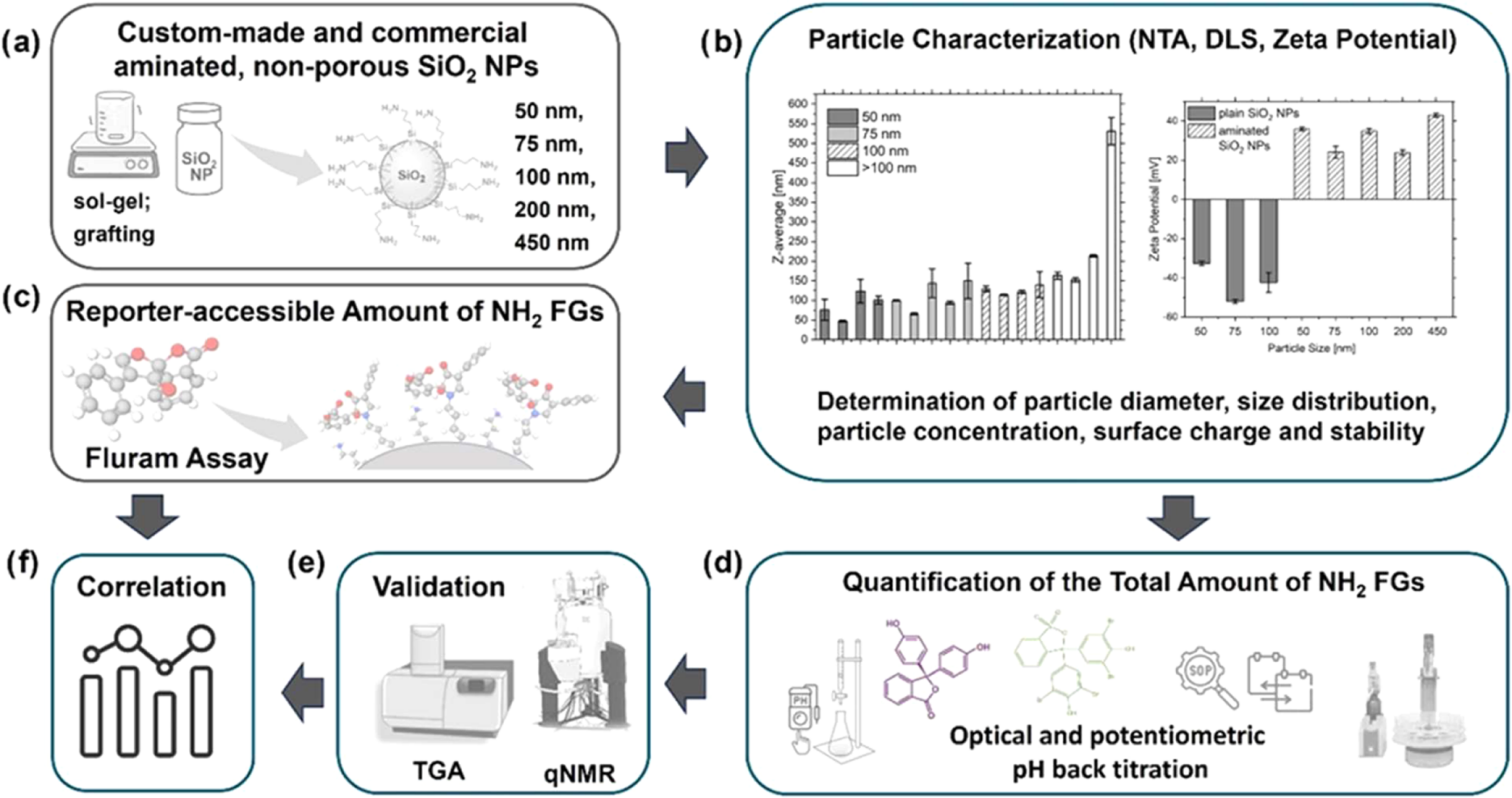
Overview of the workflows utilized for the characterization of the differently sized custom-made and commercial aminated SiO_2_ NPs, shown in panel (a). The synthesis and amination steps of the custom-made SiO_2_ NPs are shown in detail in Scheme S1 in the Supporting Information (SI). (b) Basic particle characterization with size distribution and zeta potential measurements, (c) Fluram assay providing the reporter-accessible amount of surface amino FGs, paralleled by (d) the NM-adapted pH-dependent optical and potentiometric back-titration, yielding the total amount of (de)­protonable FGs. This method was subsequently validated by (e) cross-comparison with qNMR and TGA, measuring the total amount of surface amino FGs and the total amount of organic coatings. (f) Correlation of all methods employed for surface FG screening and quantification to underline the advantages of a multimethod characterization approach.

### 3.1 NM-Adapted Potentiometric Back-Titration

Next, the applicability of a simple pH back-titration to quantify the amount of (de)­protonable surface FGs was explored for differently sized aminated SiO_2_ NPs using and comparing electrochemical (potentiometry) and optical (pH indicator) read out. Potentiometry, which measures nondestructively and fast the electrical potential of a solution between a reference and an indicator electrode, provides information about the concentration of specific ions or the chemical composition of the solution. It is commonly employed in analytical chemistry for determining the concentration of various ions in solution, e.g., in environmental monitoring, clinical analysis, and food testing.^52^ Advantages of this method are a minimum sample preparation and very inexpensive instrumentation. Electroanalytical methods such as potentiometric techniques can also aid in NM characterization in terms of surface reactivity and charge distribution. NM surface analysis, however, requires method adaptation to consider possible NM-related sources of uncertainty and method-inherent drawbacks such as the previous mentioned limited potential to distinguish between different surface FGs by conductometry. Contrary, potentiometric pH titrations could allow to discriminate between FGs varying in p*K*_a_.^53^

#### 3.1.1 Method Development & Optimization

For method development and optimization, we first assessed the applicability of pH back-titration methods previously reported for aminated SiO_2_ NPs of different sizes.^43,54,55^ These approaches were adapted from the SEARS titration used to determine the specific surface area and quantifying the number of silanol groups on SiO_2_ NPs surfaces, which involves the measurement of the adsorption of hydroxyl ions in a controlled pH range, typically between 4 and 9.^56,57^ In a pH back-titration, aminated SiO_2_ NPs are incubated with a known amount of an HCl solution of defined concentration, in our case at a pH > 3.5 (Figure S8 in the SI), to prevent particle dissolution, leading to the protonation of the surface amino FGs, followed by the (back)­titration with a NaOH solution of known concentration (SI, Figure S9). The volume of NaOH required to reach the equivalence point of about 7.0 provides the number of (protonated) amino FGs in the dispersion. With the mass of the used particles, and the (average) particle surface area, derived from DLS or NTA, the total amount of surface amino FGs per particle can be calculated. With this back-titration approach and the chosen starting point (pH > 3.5), a discrimination between different amino groups (p*K*_a_ of 7.2–7.5) and silanol groups (p*K*_a_ of 4.2–4.5) is not feasible anymore. This, however, barely affects the quantification of the amino FGs induced by surface grafting as confirmed by control experiments with nonaminated SiO_2_ NPs (SI, Figure S8).

Optical and potentiometric back-titration studies (Figure 2 and more detailed in Figures S8 and S9 in the SI) were first performed for commercial 450 nm (micro450) and 120 nm sized (NC-120) aminated SiO_2_ NPs, following published methods, that were, however, not suitable for our purpose, making an optimization necessary. Parameters thus explored included acid and base concentration, as well as the detection method, including for optical readout the pH indicator dyes used. Thereby, we focused on the p*K*_a_ values of the indicator dyes, and the ease of detecting the equivalence point by the indicator color change. The most precise results were obtained for a manual titration using 0.001 M HCl and 0.001 M NaOH. For the optically monitored pH titrations, the often-chosen indicator dye phenolphthalein with its p*K*_a_ of around 9.3 and the color change from colorless to pink in the pH range of 8.3 to 10.0 was not well suited for the detection of the equivalence point of the aminated SiO_2_ NPs of about 7.0–7.5 (Figure 2a). Better suited was the indicator bromothymol blue (BTB), which displays a strong color change from yellow over green to blue under acidic, neutral, and basic conditions, easily detectable by naked eye. BTB has also a p*K*_a_ value of 7.1, that ideally matches the end point for this reaction involving weak acids and bases near neutral pH. More importantly, the strong scattering of aqueous dispersions of SiO_2_ NPs with sizes ≥50 nm strongly complicated the visual detection of the pH-induced change in indicator color. This favors overtitration even for small SiO_2_ NPs, increases the uncertainty of optical detection for larger SiO_2_ NPs, and can introduce a virtual size dependence.

**Figure 2:**
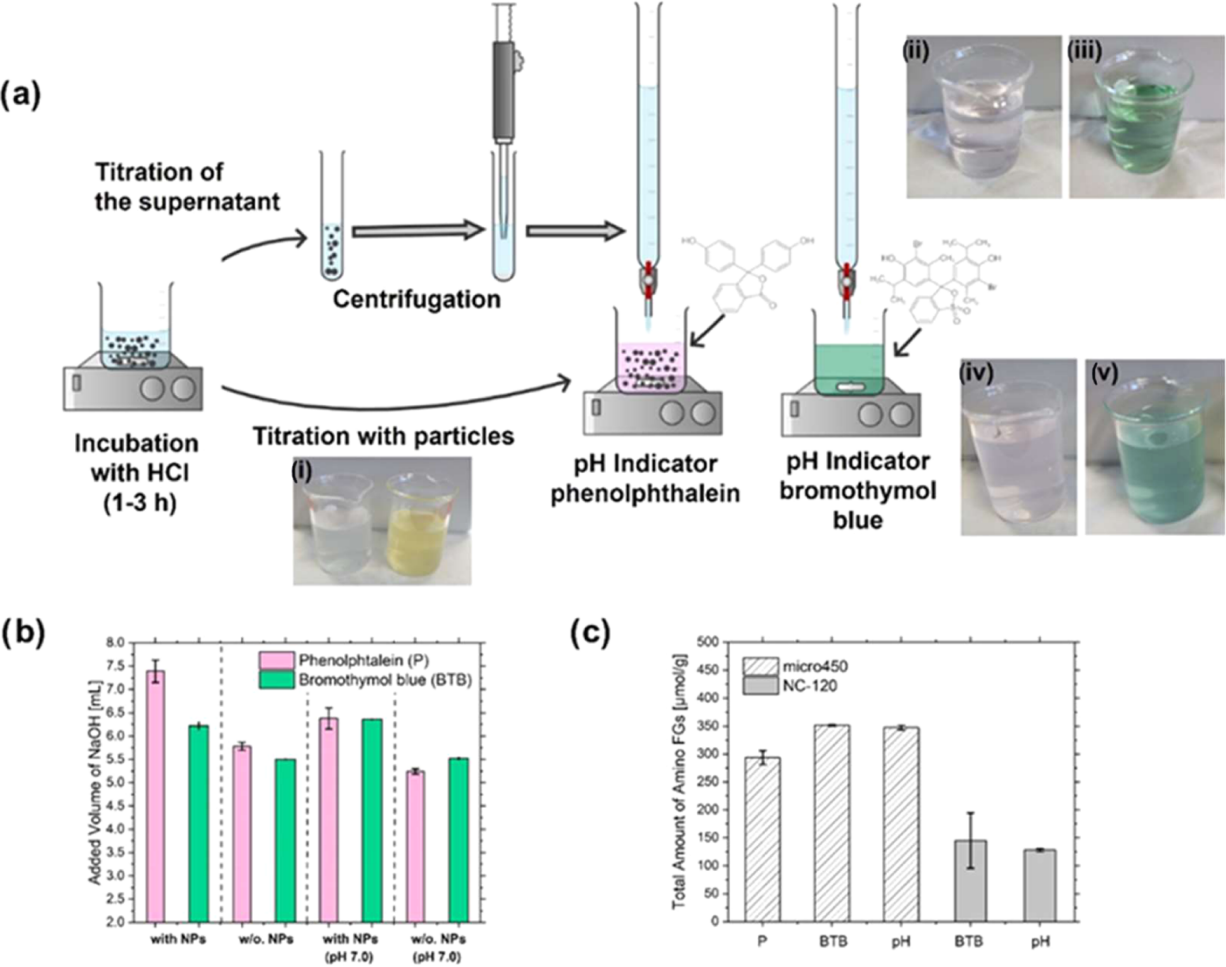
Overview of the workflow on titration with optical detection using the indicator dyes phenolphthalein (P) and bromothymol blue (BTB) and the measurement with or without (w/o) particles (a); inset (i) photograph of the particle sample with the two indicator dyes at the starting pH after incubation with HCl; (ii) photograph of the titration end point with P and w/o particles; (iii) photograph of the titration end point with BTB and w/o particles; (iv) photograph of the titration end point with P and particles; (v) photograph of the titration end point with BTB and particles. (b) Comparison of the different measurement approaches (pH indicators and separation of particles) and their influences on added volumes of NaOH. (c) Quantification of amino FGs for micro450 and NC-120 with the visually detection by pH indicator (P and BTB) and the potentiometric detection (pH).

The more sensitive and better reliable potentiometric titration with a glass-membrane pH electrode for the detection of the equivalence point, which circumvents the individual judgment of color, was hampered by the interaction of the particles with the electrode, increasing the time for establishing an equilibrium between the electrode and the solution. This affects the sensitivity and accuracy of the pH measurements, and leads to rapid electrode aging, requiring frequent displacement of the pH electrode. To circumvent NP-inherent sources of uncertainty, we subsequently refined the pH back-titration method by incubating a known amount of the previously dried aminated SiO_2_ NPs with a known amount of HCl, followed by titration of the supernatant with NaOH after particle removal. This approach, where the amount of HCl in the supernatant consumed by surface amino FG protonation is quantified by titration with NaOH, is highlighted in Figure 3 (top panel) and in Figure S9 in the SI.

**Figure 3:**
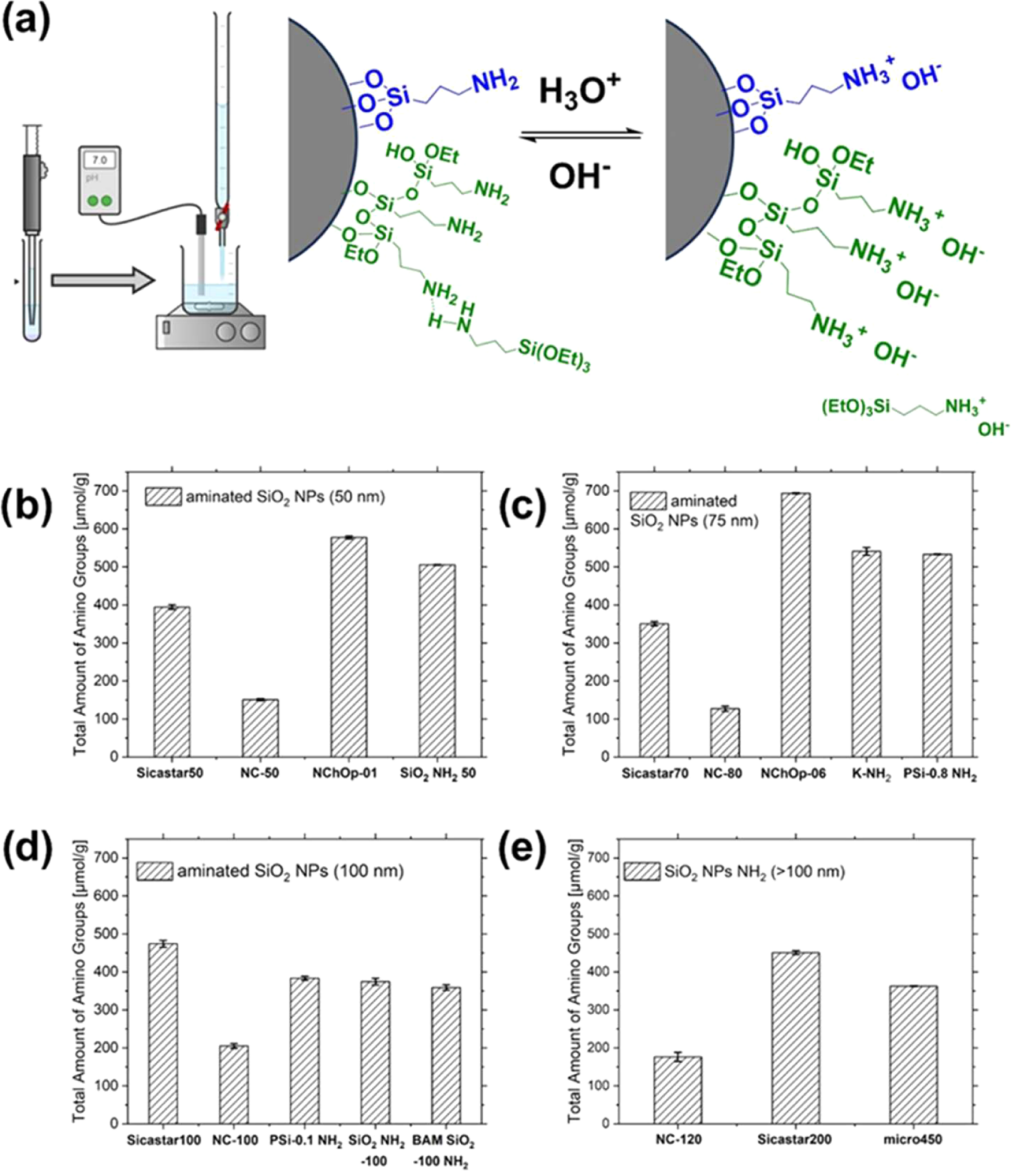
(a) Principle of the potentiometric back-titration exploiting the protonation/deprotonation of amino-modified SiO_2_ NPs grafted with a monolayer (blue) or multilayer (green) of APTES first incubated in a water/HCl mixture to protonate the amino FGs and followed by titration of the supernatant with NaOH after particle removal by centrifugation. Results of the potentiometric back-titration obtained by one operator for (b) 50 nm SiO_2_ NPs, (c) 75 nm SiO_2_ NPs, (d) 100 nm SiO_2_ NPs, and (e) >100 nm SiO_2_ NPs from different sources, varying in the degree of amination. The relative standard deviations (RSDs) of the manually performed potentiometric back-titration was for most of the measurements <3%, only NC-120 showed higher RSD (7%), confirming the reliability and robustness of our optimized workflow.

Using the exact volumes of HCl used and NaOH consumed, along with the mass of the dried particles utilized for the initial incubation step, and the (average) particle surface area, derived from DLS or NTA, decreased the uncertainty potential for the calculation of the total amount of surface amino FGs. The measurement uncertainty can be further reduced by using particle diameters derived from electron microscopy (EM) measurements instead of the number-based hydrodynamic diameters. However, the use of EM makes offline measurements necessary. Overall, the quantification of the amino FGs with optical detection is faster but less accurate, while the potentiometric detection is more precise but more time-consuming. The rate-determining step in the potentiometric method is the adjustment of the pH value or electrode potential, which can take up to 600 s per step. Our optimized workflow of the potentiometric back-titration method was then applied for FG screening and quantification studies of 50, 75, 100, and >100 nm aminated SiO_2_ NPs, chosen to consider the labeling and stability of SiO_2_ NPs with mono- and multilayers of APTES as the amount of surface amino FGs can also considerably vary for aminated SiO_2_ NPs of the same size commercialized by different suppliers. The results obtained for the aminated SiO_2_ NPs of different sizes are summarized in Figure 3 in panels b–e.

Apparently, in most cases, the amount of surface amino FGs obtained for the commercial and custom-made SiO_2_ NPs exceeds the number of APTES molecules required for the formation of a monolayer on the particle surface (Tables S1–S3 in the SI). As shown in Figure 3a, depending on the amount of amino silane used for SiO_2_ NP surface grafting, an amino silane such as APTES can form both monolayers (blue) and multilayers (green) with stable covalent bonds on the particle surfaces through hydrolysis of the ethoxy groups. The formation of a monolayer is typically achieved under reaction conditions which ensure that the APTES molecules are evenly distributed and form a lateral siloxane network with the amino groups pointing away from the particle surface. However, under less controlled conditions or for higher concentrations, APTES can polymerize and form multilayers with covalently and noncovalently bound APTES molecules. In addition, hydrogen bonds can be formed between APTES molecules, which depend on the amount of water present during the grafting step. This can occur both within a single layer and between layers.

Next, we studied the influence of purification steps during sample preparation on the uncertainty of our back-titration method by performing pH titrations of different particles that were or were not centrifuged prior the drying and weighing and the incubation in HCl. A comparison of the results shown in the SI Figure S10 revealed a significant influence of sample purification which is ascribed to the presence of released noncovalently bound molecules such as amino silane molecules, surfactants used during particle preparation and/or acting as stabilizers and acetate ions from the buffer employed by the manufacturer for particle storage which also contain (de)­protonable FGs. Hence, such purification steps and the control of the supernatant for the presence of released amino silane molecules can provide valuable information on the surface chemistry of the aminated SiO_2_ NPs, which can be relevant for particle long-term stability and subsequent labeling reaction. Such control measurements are therefore recommended for all surface functionalized NMs, where similar effects could occur. To ensure the accuracy of our back-titration method, we subsequently included a purification step into our workflow for sample preparation.

Subsequently, we determined the sensitivity of our optimized potentiometric back-titration by quantifying the amount of surface amino FGs on 80 nm aminated SiO_2_ NPs, prepared by postsynthetic grafting of one SiO_2_ core with different amounts of APTES, i.e., 1–25 equiv of APTES. As shown in Figure 4a, for APTES concentration <5 equiv no significant differences were observed for higher APTES concentrations >10 equiv a plateau was reached, that indicates the maximum amount of addition of APTES. DLS and zeta measurements show some agglomeration of batch 4 that is also visible in the titration result with the highest values. This demonstrates that our potentiometric back-titration method is suited as a simple and fast method to screen the particle surface chemistry and density of FGs for synthesis optimization.

**Figure 4:**
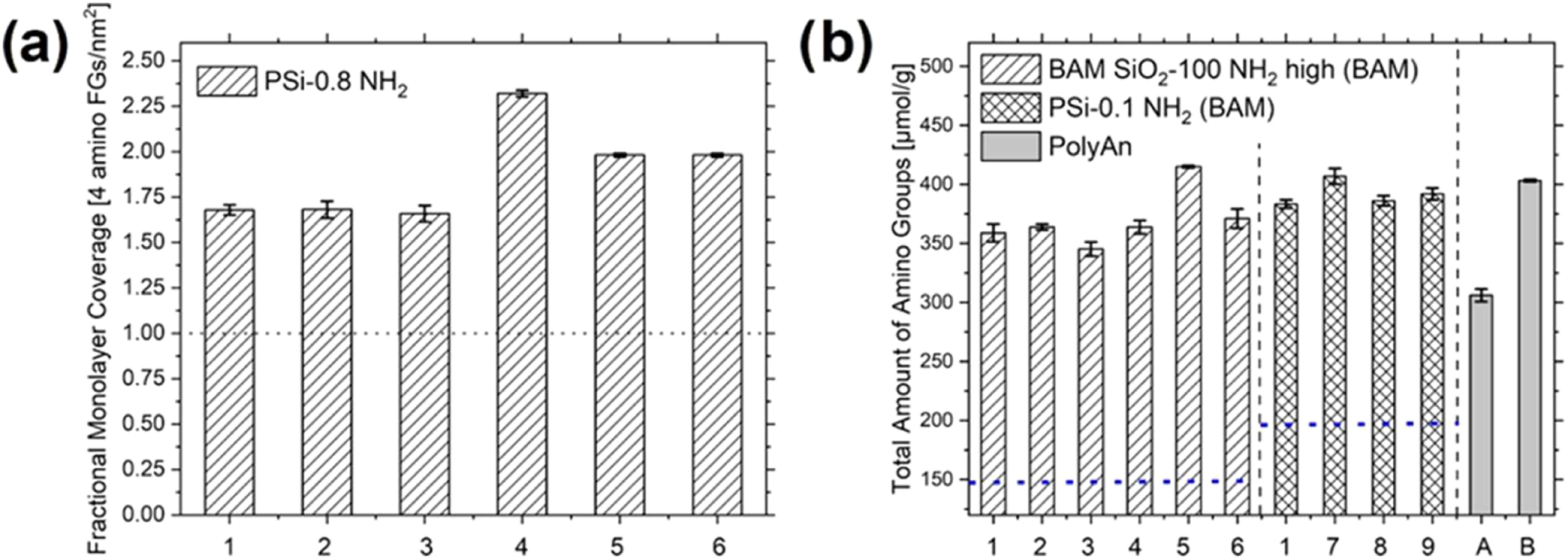
(a) Using the potentiometric back-titration method for synthesis optimization by screening 80 nm sized SiO_2_ NPs after grafting with different amino surface FG amount; and (b) reproducibility and robustness of the pH titration method studied with different operators (1–9) using custom-made BAM SiO_2_-100 NH_2_ high (A) and commercial PSi-0.1 NH_2_ (B) in two different laboratories. The dotted lines indicate a theoretical monolayer of APTES.

#### 3.1.2 Method Robustness & Bilateral Study

To assess the reliability and robustness of our titration workflow, we assessed possible sources of uncertainty of our potentiometric back pH titration method including the influence of lab equipment for differently sized SiO_2_ NPs, using our previously optimized protocols and workflows (detailed in the SI, Table S4). To minimize potential interlaboratory differences originating from, e.g., instrumentation, instrument calibration, and environmental conditions, prior to the laboratory comparison, we assured comparable environmental conditions such as room temperature, the usage of chemical of comparable quality and purity, and comparable instrument calibrations that could have an impact on relative standard deviations (RSDs). In addition, protocols for the performance of the potentiometric back-titration were provided by BAM in advance and discussed. Next, we first explored the influence of the operator on the accuracy of the manually performed titration step for representatively selected custom-made BAM SiO_2_-100 NH_2_ and commercial PSi-0.1 NH_2_ in our laboratory to assess the reliability and robustness of our workflow. Thereby, the sample preparation was performed by a well-trained operator while the potentiometric pH titration was carried out by 8 differently trained operators after a brief introduction to the use of the calibrated pH meter. As shown in Figure 4b, this led to RSD values of 6.45% (BAM SiO_2_-100 NH_2_ high) and 2.65% (PSi-0.1 NH_2_). A well-trained operator (1) can achieve RSDs of <2.00%. Subsequently, we performed a bilateral comparison for selected aminated SiO_2_ NPs (see Figure 4b gray bars, and in SI Figure S11) with an industrial producer of surface functionalized NMs, the company PolyAn. This company, like many SMEs, uses optical assays and conductometry to determine the amount of surface FGs for production and quality control of their products. This revealed RSDs of 12.15 and 1.62% for BAM SiO_2_-100 NH_2_ high and PSi-0.1 NH_2_, respectively, for the PolyAn operator. Particularly important seemed to be the use of a high quality and sensitive pH electrode and a standardized sample preparation protocol as revealed by a comparison of the results obtained for particle drying for 1–2 h and overnight at 80 °C for 50–200 nm Sicastar SiO_2_ NPs (Figure S11). Particle drying should be performed at 80 °C for at least 4–6 h. Principally, the drying temperatures and periods could be further fine-tuned to optimize the analysis time, which was, however, beyond the scope of this study.

### 3.2 Method Comparison with qNMR & TGA and an Optical Assay

Finally, both detection methods utilized for pH back-titrations were compared and validated by cross-comparison with qNMR and TGA (Figures S12 and S13 in the SI). The particles were prepared similar for TGA, qNMR, and titration measurements including a purification step to separate free amine and other noncovalently bound molecules by centrifugation or dialysis, drying overnight, followed by weighing and redispersion (titration/qNMR) or direct measurements (TGA). The highest amount of particles is required for qNMR measurements, with a minimum of 30 mg needed for a triplicate experiment, whereas titration and TGA measurements require only 15 mg for the same. For titration measurements the particles were redispersed and incubated with HCl, separation of the supernatant by centrifugation and was used with a specific volume for the titration step. For qNMR measurements the particles were redispersed and incubated with NaOD/D_2_O to dissolve the particles, followed by addition of ultrapure maleic acid as an internal standard. Both methods need several preparation steps introducing different uncertainties. The analysis of the qNMR data seems to be more complex and error prone compared to the relatively simple analysis of the titration measurements. qNMR requires expensive instrumentation and well-trained operators. In addition, the reliability and accuracy of qNMR measurements strongly depend on several factors. Main sources of uncertainty are the accuracy of the sample preparation procedure, particularly the drying, weighing, and dissolution steps, the NMR data acquisition parameters, and the data evaluation procedures such as baseline correction and integration methods.^28^ Analysis of the TGA measurements, which do not require a particle dissolution step, reveals that the achievable measurement uncertainties are affected by drying and weighing and the possible presence of nonaminated surfactants or stabilizers.

A comparison of the back-titration approaches performed by two operators and validated with qNMR and TGA for NC-120 is shown in Figure 5a. The results reveal matching trends for the potentiometric back-titration and qNMR, underlining the correlation between the results provided by both methods, that measure different measurands. The slight differences between the measurements originated from the different chemo-selectivity of both methods. The former method nonselectively detects all protonable FGs present in solution, which can favor an overestimation. Contrary, qNMR chemo-selectively measures the amount of amino FGs or amino silane molecules released upon particle dissolution, by calculating the amount of FGs using an internal standard and the signals of the aminopropyl group.

**Figure 5:**
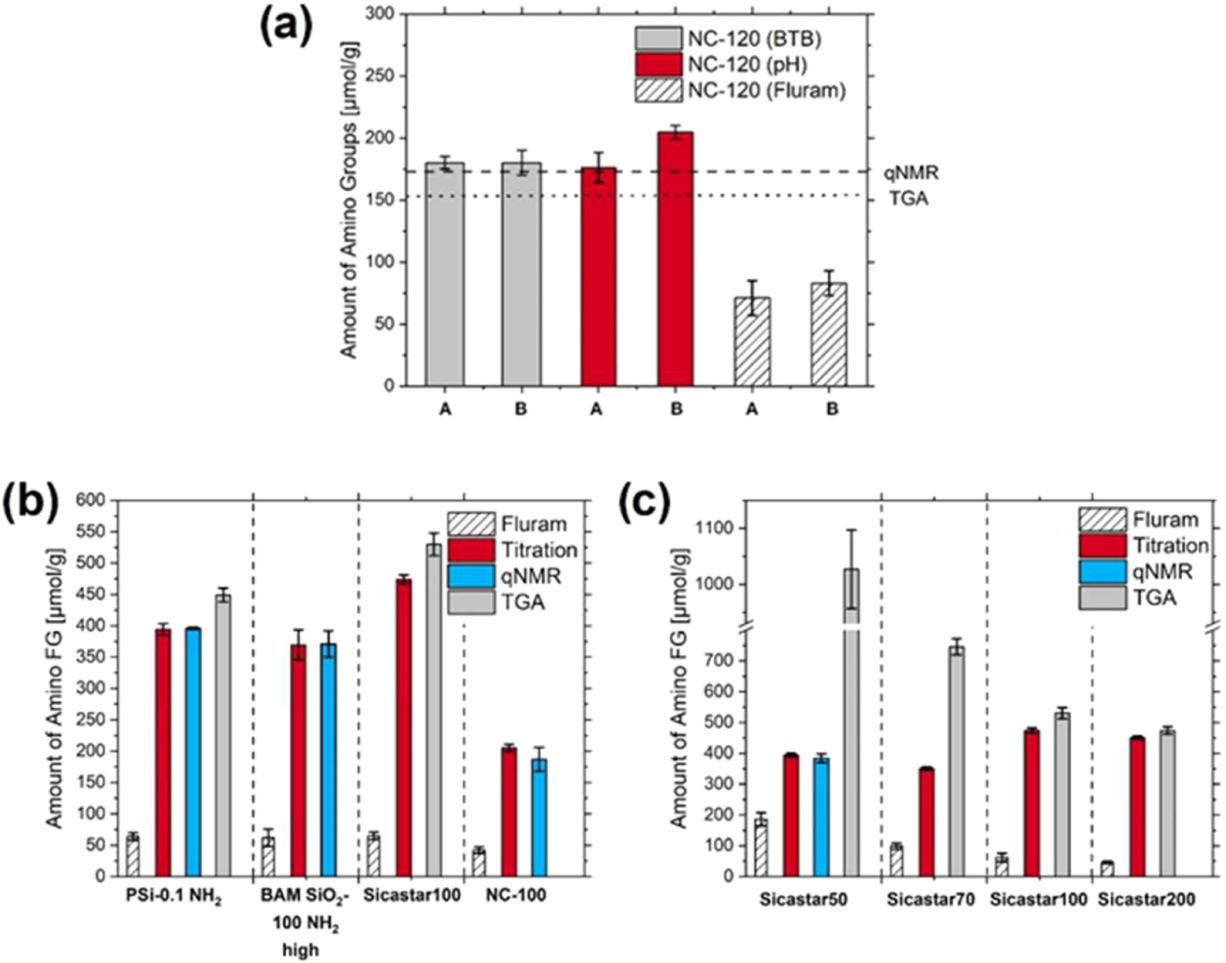
(a) Comparison of the two back-titration approaches with optical detection (BTB, gray bars) and potentiometric (pH, red bars) measurements performed by two operators (A and B) for the total amount of amino FG with an optical Fluram assay for a reporter-accessible amount of amino FGs and their validation with qNMR (dashed line) and TGA (dotted line) measurements. Correlation of potentiometric titration with TGA and with qNMR for method validation by cross-comparison for different 100 nm aminated particles (b) and different sized aminated Sicastar particles (c).

Principally, a potentiometric pH titration enables to distinguish between different (de)­protonable FGs varying in p*K*_a_, but as previously mentioned, since we used a back-titration approach for our particles, changes in the point of zero charge of different ionic groups cannot be exploited anymore for a discrimination. TGA measurements of NC-120 showed slightly lower but comparable results. In contrast, the TGA results obtained for PSi-0.1 NH_2_ and the Sicastar particles revealed significantly higher values (SI, Figure S12 and Table S5). The size of the measured mass points to the presence of different groups or molecules on the particle surface, which can also explain the negative zeta potentials of these particles (SI Figure S7).

#### 3.2.1 Comparison with the Fluram Assay

Finally, the amount of surface amino FGs was determined with a semiautomated optical Fluram assay. As mentioned before, optical assays such as the Fluram assay always yield an assay-specific number of FGs interacting and reacting with the chosen signal-generating dye reporter of a given size, spatial requirement, and charge. This number, which is also affected by the size and surface morphology of the surface-functionalized particle system, should be hence significantly lower than the results obtained by our potentiometric back-titration.^6,37,39^ As revealed in Figure 5b,c, and in the SI (Figures S14–S17, and Table S6), depending on the particle size and preparation of the aminated SiO_2_ NPs, the differences between the total and reporter-accessible amount of surface amino FGs can significantly vary. Table S6 in the SI also highlights the material-specific influence on the amount of reporter-accessible amino FGs by comparing the total amount and the reporter-accessible amount of amino FGs determined for the various custom-made and commercial SiO_2_ NPs with the potentiometric back-titration and the Fluram assay. This clearly shows that optical assays are useful tools for measurements where fast information on the relative amount of surface FGs is sufficient, e.g., for synthesis screening and for the quality control of surface functionalized particles and NMs where changes in the relative amount of surface FGs for the same material need to be frequently measured. Ideal areas of application are homogeneity and stability monitoring studies. Optical assay should be, however, used with care for the comparison of different surface functionalized particles.

### 3.3 Automated vs Manual Titration

The accuracy and reproducibility of our manual back-titration is affected by the experience and technical skills of the operator.^58,59^ Additionally, the manual titration can be time-consuming. The time required for triplicate measurements covers the sample preparation, including purification steps, sample drying (waiting time), particle weighing and redispersion, and the preparation of the titrants (around 2–3 h), the determination of the titer in triplicate (2 h), incubation of the dried particles with HCl (1–3 h), and sample titration in triplicate. The different operators involved in this study the pH titrations, needed 45 to 100 min for the manual titration. Hence, on a working day of 8 h, one or two titrations can be run in triplicate (Figure S19 in the SI). This time can be further reduced, which was, however, beyond the scope of this study. Even with a simple automation approach relying on a commercial titrator, the hands-on titration time could be significantly reduced. In addition, automated titrators, which employ a very precise motor-driven piston buret to dispense the titrant in extremely small increments (0.010 mL) make such titrations more reliable and reproducible. We subsequently tested our titration approach with two types of commercially available automated titrators, a pH module with automated dosing units, and a more advanced automatic sample processor with a built-in membrane pump for the processing of samples in series. These devices performed the semiautomated back-titration of BAM SiO_2_-100 NH_2_ using 0.001 M NaOH/HCl with RSDs of 7.90 and 1.83%, respectively. The size of the resulting RSD values was largely determined by the sensitivity of the pH electrode (SI, Figure S18).Overall, with this simple automation, the hands-on preparation time for titer determination and the titration step could be significantly reduced from an average of 5–7 h to 2–3 h Depending on the selected program and increment size, automated titration requires only 0.75–1.5 h per sample while maintaining high accuracy. With semiautomation, 2–3 samples can be processed per day, and with full automation, the titration method can theoretically be performed continuously (24/7).

## 4 Conclusion & Outlook

The reproducible synthesis of engineered nanomaterials (NMs), NM quality control, stability, and aging studies as well as toxicity and exposure studies require a toolbox of simple and validated methods, that provide quantitative information on NM surface chemistry and functional groups (FGs) with broadly available and cost-efficient instrumentation and can be ideally automated with affordable commercial tools. Thereby, increasing regulatory requirements and safety concerns can be more effectively addressed as the surface chemistry largely controls NM dispersibility, stability, processability, and interaction with the environment. As an expansion of our NM surface analysis toolbox, focusing on the development and validation of such simple methods to support the nanotechnology community and small and medium sized enterprises (SMEs), we developed a NM-adapted pH titration method (Figure 6) for measuring the total number of (de)­protonable FGs on commercial and custom-made aminated silica nanoparticles (SiO_2_ NPs) varying in size and amino FG density. By examining potentiometric and optical readout with indicator dyes, critical steps for method accuracy and reliability could be identified such as the need to remove the dispersed NMs prior to the pH back-titration, to circumvent undesired and material-specific NM interferences, a standardized sample preparation workflow including washing and drying steps of the removed NMs prior to NM weighing as well as the crucial choice of an indicator dye with a p*K*_a_ matching the equivalence point of the back-titration of about 7.0, which undergoes a strong color change, or a sensitive pH electrode. The later provides a higher sensitivity and is less error prone. The accuracy and robustness of these stepwise optimized workflows were then assessed for the potentiometric back-titration and two exemplary chosen aminated SiO_2_ NP samples, by several operators at BAM and at a SME producing surface functionalized NMs to derive achievable relative standard deviations (RSDs). A well-trained operator can perform one or two potentiometric back-titration measurements in triplicate within a working day of 8 h with a RSD of <2.00%. In addition, the automation of this pH titration was presented, using two different automated titration setups. These measurements, which could also be integrated into online analysis workflows, provided a higher accuracy compared to less experienced operators. The reliability of this NM-adapted pH titration method was confirmed by method cross-comparison with traceable quantitative nuclear magnetic resonance spectroscopy (qNMR), chemo-selectively measuring the total amount of amino FGs using the NMR signals of the surface grafted amino silane, and thermogravimetric analysis (TGA), providing the total amount of the organic NM coating. Comparing our pH titration data with the amount of reporter-accessible amino FGs, obtained with the Fluram assay, previously automated by us, which presents an assay- and reporter-specific number, highlights the importance of measuring both quantities for surface-functionalized NMs for optimum quality control and NM applications.

**Figure 6:**
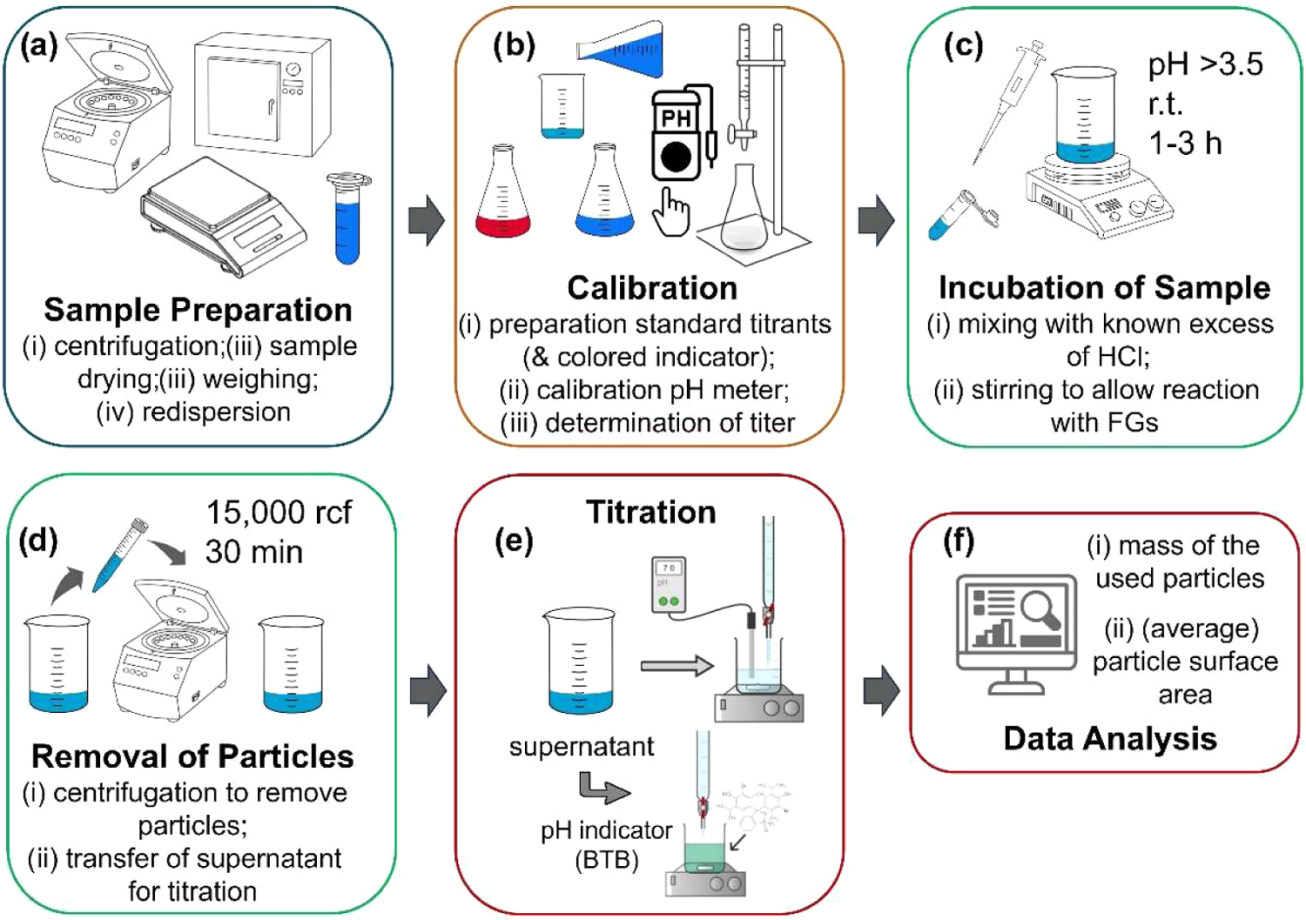
Overview of the workflow for the back-titration method with its single steps (a) sample preparation; (b) calibration; (c) incubation of the particle sample; (d) removal of particles by centrifugation; (e) potentiometric titration or optical detection using the indicator dye bromothymol blue (BTB); and (f) data analysis with the mass of the used particles and the (average) particle surface area, derived from DLS or NTA, to calculate the total amount of surface amino FGs. A gantt chart visualizing the estimated time of the manually performed titration is shown in the SI in Figure S19I.

In summary, the cost-efficient and automatable potentiometric back-titration method presented here is a universal method suitable many different NMs and particle systems varying in chemical composition, requiring only that the particles are stable under the different pH values used for the potentiometric back-titration. As solely the supernatant is measured, the particle matrix does not play a role. Our multimethod approach for surface FG screening, quantification, and monitoring using this cost-efficient pH titration together with a semiautomated optical assay can provide important information on NM batch-to-batch reproducibility, NM stability, and for additional application-relevant labeling steps as well as for NM toxicity and exposure studies. It also allows to compare surface functionalized NMs from different sources and/or produced by different synthetic routes or with different chemicals. This is, e.g., significant for broadly used NM systems such as custom-made or commercial nonporous aminated SiO_2_ NPs, where the amount of surface amino FGs can considerably vary from batch to batch, yielding mono- or multilayer amino silane surface structures, depending on the producer, i.e., the synthetic method, ratio of reagents, and amino silane applied for surface grafting as is shown in this study. These variations in SiO_2_ NP surface chemistry can possibly affect further processing steps and particle long-term stability.

Currently, we are assessing the applicability of our potentiometric titration method for synthesis screening, additional particle types, and other FGs such as carboxyl groups. In addition, we are exploring the fine-tuning of the pH range used for our pH back-titration method to more effectively exploit differences in the p*K*_a_ values of common surface FGs, thereby improving the chemo-selectivity of the method. In this context, we meanwhile applied our potentiometric back-titration method to in-house synthesized aminated polystyrene NPs as revealed in the SI (Figure S20) and to commercial carboxylated SiO_2_ NPs by adjusting the titration conditions to match the acid–base characteristics of the respective surface functionality. Strategies for p*K*_a_ differentiation explored by us in the future will include the resolution of functional groups by p*K*_a_, e.g., by performing potentiometric titrations at lower ionic strength or segmented titration curves. Also, we will further develop our automation approaches for combined NM surface analysis with focus on different optical assays and signal generation strategies. In the future, our continuously growing platform of simple, validated, and automatable methods for FG screening, quantification, and monitoring, now expanding by a NM-adapted versatile pH titration method, will simplify quality control of NM production processes and stability studies and speed up the generation of large data sets for NM grouping for sustainable and safe­(r)-by-design approaches.

## Supplementary Material



## Notes

Detailed information on the particle synthesis and characterization by DLS, NTA, and zeta potential measurements, as well as the quantification of amino FGs with optical and potentiometric back-titration, and the validation with qNMR, TGA, and Fluram assay (PDF)

## References

[1] Abram S. L., Tavernaro I., Johnston L. J., Zou S., Resch-Genger U. (2025). Nanoscale
reference and test materials for the validation of characterization
methods for engineered nanomaterials — current state, limitations,
and needs. Anal. Bioanal. Chem..

[2] Kokila G. N., Mallikarjunaswamy C., Ranganatha V. L. (2024). A review on synthesis and applications
of versatile nanomaterials. Inorg. Nano-Metal
Chem..

[3] Labuda J., Barek J., Gajdosechova Z., Goenaga-Infante H., Johnston L. J., Mester Z., Shtykov S. (2023). Analytical
Chemistry
of Engineered Nanomaterials: Part 1. Scope, Regulation, Legislation,
and Metrology (IUPAC Technical Report). Pure
Appl. Chem..

[4] Gubala V., Johnston L. J., Krug H. F., Moore C. J., Ober C. K., Schwenk M., Vert M. (2018). Engineered
nanomaterials and human
health: Part 2. Applications and nanotoxicology (IUPAC Technical Report). Pure Appl. Chem..

[5] Tavernaro I., Dekkers S., Soeteman-Hernández L. G., Herbeck-Engel P., Noorlander C., Kraegeloh A. (2021). Safe-by-Design
part II: A strategy
for balancing safety and functionality in the different stages of
the innovation process. NanoImpact.

[6] Geißler D., Nirmalananthan-Budau N., Scholtz L., Tavernaro I., Resch-Genger U. (2021). Analyzing
the surface of functional nanomaterials—how
to quantify the total and derivatizable number of functional groups
and ligands. Microchim. Acta.

[7] Dekkers S., Wijnhoven S. W. P., Braakhuis H. M., Soeteman-Hernandez L.
G., Sips A. J. A. M., Tavernaro I., Kraegeloh A., Noorlander C. W. (2020). Safe-by-Design
part I: Proposal for nanospecific human
health safety aspects needed along the innovation process. NanoImpact.

[8] Bai Y., Hao M., Ding S., Chen P., Wang L. (2022). Surface Chemistry
Engineering
of Perovskite Quantum Dots: Strategies, Applications, and Perspectives. Adv. Mater..

[9] Hirmer, A. ; Märkl, S. ; Schroter, A. ; Hirsch, T. In Surface engineering of small and bright upconversion nanoparticles providing chemical and colloidal stability in biological media, Proceedings Nanoengineering: Fabrication, Properties, Optics, Thin Films, and Devices, 2020; p 114670X.

[10] Manoj G. M., Shalini M., Thenmozhi K., Ponnusamy V. K., Hari S. (2024). Recent advancements in the surface modification and functionalization
of magnetic nanomaterials. Appl. Surf. Sci.
Adv..

[11] Díez-Pascual A.
M. (2022). Surface
Engineering of Nanomaterials with Polymers, Biomolecules, and Small
Ligands for Nanomedicine. Materials.

[12] Austin L.
A., Mackey M. A., Dreaden E. C., El-Sayed M. A. (2014). The optical, photothermal,
and facile surface chemical properties of gold and silver nanoparticles
in biodiagnostics, therapy, and drug delivery. Arch. Toxicol..

[13] Holzinger M., Le Goff A., Cosnier S. (2014). Nanomaterials
for biosensing applications:
a review. Front. Chem..

[14] Kumar H., Yan M. (2025). Quantification of Nanomaterial
Surfaces. Mater.
Interfaces.

[15] Smith A. M., Johnston K. A., Crawford S. E., Marbella L. E., Millstone J. E. (2017). Ligand
density quantification on colloidal inorganic nanoparticles. Analyst.

[16] Jayawardena H. S. N., Liyanage S. H., Rathnayake K., Patel U., Yan M. (2021). Analytical
Methods for Characterization of Nanomaterial Surfaces. Anal. Chem..

[17] Lopinski G. P., Kodra O., Kunc F., Kennedy D. C., Couillard M., Johnston L. J. (2025). X-ray photoelectron
spectroscopy of metal oxide nanoparticles:
chemical composition, oxidation state and functional group content. Nanoscale Adv..

[18] Bennet F., Müller A., Radnik J., Hachenberger Y., Jungnickel H., Laue P., Luch A., Tentschert J. (2020). Preparation
of Nanoparticles for ToF-SIMS and XPS Analysis. J. Vis. Exp..

[19] Baer D. R., Gaspar D. J., Nachimuthu P., Techane S. D., Castner D. G. (2010). Application
of surface chemical analysis tools for characterization of nanoparticles. Anal. Bioanal. Chem..

[20] Zeng B., Palui G., Zhang C., Zhan N., Wang W., Ji X., Chen B., Mattoussi H. (2018). Characterization of the Ligand Capping
of Hydrophobic CdSe–ZnS Quantum Dots Using NMR Spectroscopy. Chem. Mater..

[21] Zhang C., Palui G., Zeng B., Zhan N., Chen B., Mattoussi H. (2018). Non-Invasive Characterization of
the Organic Coating
of Biocompatible Quantum Dots Using Nuclear Magnetic Resonance Spectroscopy. Chem. Mater..

[22] Kroupa D. M., Voros M., Brawand N. P., McNichols B. W., Miller E. M., Gu J., Nozik A. J., Sellinger A., Galli G., Beard M. C. (2017). Tuning colloidal
quantum dot band
edge positions through solution-phase surface chemistry modification. Nat. Commun..

[23] Smith A. M., Marbella L. E., Johnston K. A., Hartmann M. J., Crawford S. E., Kozycz L. M., Seferos D. S., Millstone J. E. (2015). Quantitative
analysis of thiolated ligand exchange on gold nanoparticles monitored
by 1H NMR spectroscopy. Anal. Chem..

[24] Xu J. X., Alom M. S., Fitzkee N. C. (2021). Quantitative
Measurement of Multiprotein
Nanoparticle Interactions Using NMR Spectroscopy. Anal. Chem..

[25] Huang R., Fedeli S., Hirschbiegel C.-M., Zhang X., Rotello V. M. (2024). Modulation
of Gold Nanoparticle Ligand Structure–Dynamic Relationships
Probed Using Solution NMR. ACS Nanosci. Au.

[26] Sun Y., Kunc F., Balhara V., Coleman B., Kodra O., Raza M., Chen M., Brinkmann A., Lopinski G. P., Johnston L. J. (2019). Quantification of amine functional
groups on silica nanoparticles: a multi-method approach. Nanoscale Adv..

[27] Kunc F., Balhara V., Sun Y., Daroszewska M., Jakubek Z. J., Hill M., Brinkmann A., Johnston L. J. (2019). Quantification of surface functional groups on silica
nanoparticles: comparison of thermogravimetric analysis and quantitative
NMR. Analyst.

[28] Kunc F., Nirmalananthan-Budau N., Rühle B., Sun Y., Johnston L. J., Resch-Genger U. (2021). Interlaboratory
Comparison on the Quantification of
Total and Accessible Amine Groups on Silica Nanoparticles with qNMR
and Optical Assays. Anal. Chem..

[29] Crucho C. I. C., Baleizao C., Farinha J. P. (2017). Functional Group Coverage and Conversion
Quantification in Nanostructured Silica by (1)H NMR. Anal. Chem..

[30] Almaghrabi M., Alqurshi A., Jadhav S. A., Mazzacuva F., Cilibrizzi A., Raimi-Abraham B., Royall P. G. (2023). Evaluating thermogravimetric
analysis for the measurement of drug loading in mesoporous silica
nanoparticles (MSNs). Thermochim. Acta.

[31] Zhu S., Panne U., Rurack K. (2013). A rapid method
for the assessment
of the surface group density of carboxylic acid-functionalized polystyrene
microparticles. Analyst.

[32] Alessi A., Agnello S., Buscarino G., Gelardi F. M. (2013). Raman and IR investigation
of silica nanoparticles structure. J. Non-Cryst.
Solids.

[33] Dendisová M., Jeništová A., Parchaňská-Kokaislová A., Matějka P., Prokopec V., Švecová M. (2018). The use of
infrared spectroscopic techniques to characterize nanomaterials and
nanostructures: A review. Anal. Chim. Acta.

[34] Sikora A., Bartczak D., Geißler D., Kestens V., Roebben G., Ramaye Y., Varga Z., Palmai M., Shard A. G., Goenaga-Infante H., Minelli C. (2015). A systematic comparison of different
techniques to determine the zeta potential of silica nanoparticles
in biological medium. Anal. Methods.

[35] Tavernaro I., Matiushkina A., Rother K. S., Mating C., Resch-Genger U. (2024). Exploring
the potential of simple automation concepts for quantifying functional
groups on nanomaterials with optical assays. Nano Res..

[36] Zhou H., Tourkakis G., Shi D., Kim D. M., Zhang H., Du T., Eades W. C., Berezin M. Y. (2017). Cell-free
measurements of brightness
of fluorescently labeled antibodies. Sci. Rep..

[37] Nirmalananthan-Budau N., Rühle B., Geißler D., Moser M., Kläber C., Schäfer A., Resch-Genger U. (2019). Multimodal Cleavable Reporters for
Quantifying Carboxy and Amino Groups on Organic and Inorganic Nanoparticles. Sci. Rep..

[38] Felbeck T., Hoffmann K., Lezhnina M. M., Kynast U. H., Resch-Genger U. (2015). Fluorescent
Nanoclays: Covalent Functionalization with Amine Reactive Dyes from
Different Fluorophore Classes and Surface Group Quantification. J. Phys. Chem. C.

[39] Hennig A., Borcherding H., Jaeger C., Hatami S., Würth C., Hoffmann A., Hoffmann K., Thiele T., Schedler U., Resch-Genger U. (2012). Scope and Limitations of Surface Functional Group Quantification
Methods: Exploratory Study with Poly­(acrylic acid)-Grafted Micro-
and Nanoparticles. J. Am. Chem. Soc..

[40] Laux E.-M., Behnke T., Hoffmann K., Resch-Genger U. (2012). Keeping particles
brilliant–simple methods for the determination of the dye content
of fluorophore-loaded polymeric particles. Anal.
Methods.

[41] Moser M., Schneider R., Behnke T., Schneider T., Falkenhagen J., Resch-Genger U. (2016). Ellman’s and Aldrithiol Assay
as Versatile and Complementary Tools for the Quantification of Thiol
Groups and Ligands on Nanomaterials. Anal. Chem..

[42] Xing Y., Borguet E. (2007). Specificity and Sensitivity of Fluorescence Labeling
of Surface Species. Langmuir.

[43] Hofen K., Weber S., Chan C. P. C., Majewski P. (2011). Novel titration method
for surface-functionalised silica. Appl. Surf.
Sci..

[44] Mörnstam B., Wahlund K.-G., Jönsson B. (1997). Potentiometric
Acid-Base Titration
of a Colloidal Solution. Anal. Chem..

[45] Szekeres M., Tombácz E. (2012). Surface charge
characterization of metal oxides by
potentiometric acid–base titration, revisited theory and experiment. Colloids Surf., A.

[46] Charron G., Hühn D., Perrier A., Cordier L., Pickett C. J., Nann T., Parak W. J. (2012). On the Use of pH Titration to Quantitatively
Characterize Colloidal Nanoparticles. Langmuir.

[47] Roebben G., Kestens V., Varga Z., Charoud-Got J., Ramaye Y., Gollwitzer C., Bartczak D., Geissler D., Noble J., Mazoua S., Meeus N., Corbisier P., Palmai M., Mihaly J., Krumrey M., Davies J., Resch-Genger U., Kumarswami N., Minelli C., Sikora A., Goenaga-Infante H. (2015). Reference
materials and representative test materials
to develop nanoparticle characterization methods: the NanoChOp project
case. Front. Chem..

[48] Tavernaro I., Cavelius C., Peuschel H., Kraegeloh A. (2017). Bright fluorescent
silica-nanoparticle probes for high-resolution STED and confocal microscopy. Beilstein J. Nanotechnol..

[49] Zhuravlev L. T. (1987). Concentration
of hydroxyl groups on the surface of amorphous silicas. Langmuir.

[50] Dietrich P. M., Streeck C., Glamsch S., Ehlert C., Lippitz A., Nutsch A., Kulak N., Beckhoff B., Unger W. E. S. (2015). Quantification
of Silane Molecules on Oxidized Silicon: Are there Options for a Traceable
and Absolute Determination?. Anal. Chem..

[51] Cueto-Díaz E. J., Castro-Muñiz A., Suárez-García F., Gálvez-Martínez S., Torquemada-Vico M. C., Valles-González M. P., Mateo-Martí E. (2021). APTES-Based
Silica Nanoparticles as a Potential Modifier for the Selective Sequestration
of CO2 Gas Molecules. Nanomaterials.

[52] Zdrachek E., Bakker E. (2019). Potentiometric Sensing. Anal.
Chem..

[53] Han G. E., Priefer R. (2023). A systematic review of various pK­(a)
determination
techniques. Int. J. Pharm..

[54] Jung H.-S., Moon D.-S., Lee J.-K. (2012). Quantitative
Analysis and Efficient
Surface Modification of Silica Nanoparticles. J. Nanomater..

[55] Kotsyuda S. S., Tomina V. V., Zub Y. L., Furtat I. M., Lebed A. P., Vaclavikova M., Melnyk I. V. (2017). Bifunctional silica nanospheres with
3-aminopropyl and phenyl groups. Synthesis approach and prospects
of their applications. Appl. Surf. Sci..

[56] Heinroth F., Münnekhoff R., Panz C., Schmoll R., Behnisch J., Behrens P. (2008). The Sears
number as a probe for the surface chemistry
of porous silicas: Precipitated, pyrogenic and ordered mesoporous
silicas. Microporous Mesoporous Mater..

[57] Sears G. W. (1956). Determination
of specific surface area of colloidal silica by titration with sodium
hydroxide. Anal. Chem..

[58] Lo S., Baird S. G., Schrier J., Blaiszik B., Carson N., Foster I., Aguilar-Granda A., Kalinin S. V., Maruyama B., Politi M., Tran H., Sparks T. D., Aspuru-Guzik A. (2024). Review of
low-cost self-driving laboratories in chemistry and materials science:
the “frugal twin” concept. Digital
Discovery.

[59] Peters M., Siegfried L., Kaden T. A. (1999). A fully automated pH–NMR titration
set-up for protonation studies. J. Chem. Soc.,
Dalton Trans..

