# Supplementary material for: Expanding the Toolbox of Simple, Cost-Efficient, and Automatable Methods for Quantifying Surface Functional Groups on Nanoparticles—Potentiometric Titration
Source: ACS Meas Sci Au. 2025 Aug 20;5(5):695–707. doi: 10.1021/acsmeasuresciau.5c00062 (PMC12532055; doi:10.1021/acsmeasuresciau.5c00062)
Supplement: Supplementary file 1 [file tg5c00062_si_001.pdf]

Supporting Information

Expanding the Toolbox of Simple, Cost-efficient, and Automatable Methods for Quantifying Surface Functional Groups on Nanoparticles – Potentiometric Titration

Isabella Tavernaro[1,§,*], Philipp C. Sander[1,§] Elina Andresen[1], Uwe Schedler[2], and Ute Resch-Genger[1,*]

[1]Federal Institute for Materials Research and Testing (BAM), Division Biophotonics, Richard-Willstaetter-Str. 11, 12489 Berlin, Germany.

[2]PolyAn, Schkopauer Ring 6, 12681 Berlin, Germany.

[§]I. Tavernaro and P.C. Sander contributed equally to this work

Corresponding Author

*E-mail: isabella.tavernaro@bam.de; ute.resch@bam.de

Index

1. Particle Synthesis 2

2. Particle Characterization 3

2.1 Dynamic Light Scattering....3

2.2 Nanoparticle Tracking Analysis (NTA) 7

2.3 Zeta Potential Measurements....8

2.4 Estimation of a monolayer of amino FGs....9

3. Quantification of the Total Amount of Amino Functional Groups using a pH Titration Approach..11

3.1 Determination of the titrants....12

3.2 Optical and potentiometric pH -Titration 13

3.3 Determination of the Amount of amino FGs....14

3.4 Bilateral comparison BAM and PolyAn....16

3.5 Potential Influences of the sample Preparation on the Potentiometric Back Titration....16

4. Validation with TGA and qNMR....17

4.1 TGA....18

4.2 qNMR....20

5. Fluram Assay....21

6. Automation....25

7. Adaptation to other functional surface groups and types of particles 28
8. References 29

1. Particle Synthesis

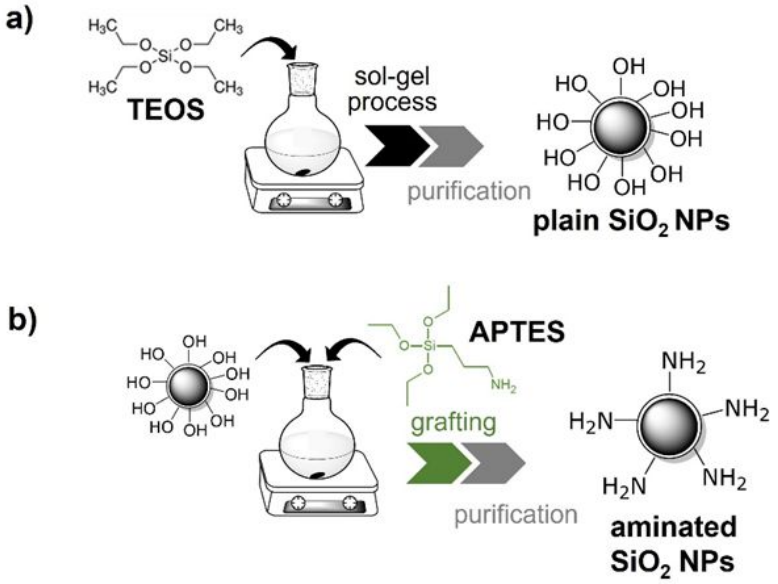

Scheme S1. Reaction scheme of a) the particle synthesis and b) amination steps of custom-made SiO_2 NPs.

BAM SiO_2-100 NH_2 high were synthesized in a two-step approach (Scheme S1), utilizing a tailored sol-gel synthesis route with tetraethyl orthosilicate (TEOS, Sigma Aldrich, Germany) as a silicon precursor, followed by amination with an excess of 3-aminopropyltriethoxysilane (APTES, abcr GmbH, Germany) in ethanol (EtOH, Labsolute, Th Geyer, Germany) using previously described methods.[1, 2]

For sensitivity studies PSi-0.8 plain SiO_2 NPs (Kisker Biotech GmbH & Co. KG, Germany, lot#: GK2881343-01) were aminated with different concentrations of APTES in EtOH, using a Carousel 6 multisynthesis device (Radleys, UK) at a temperature of 40 °C. Thereby, the SiO_2 NPs were first dispersed in absolute EtOH and degassed under argon for 30 min, then adequate amounts of APTES were added under gentle stirring. We chose an excess of 1.0-, 2.5-, 5.0-, 7.5-, 10.0-, and 25.0-times higher amounts of APTES than required for a calculated monolayer. The reaction mixture was stirred under argon atmosphere for 2 d to finalize the hydrolysis of the ethoxy groups and allow the formation of the stable siloxane groups on the particle surface.

Purification was done by precipitation via centrifugation (Hettich Rotina R380, 15,000 rcf, 30 min) and several washing steps using EtOH and MilliQ-water:EtOH mixtures. The particles were redispersed in 10 mL of EtOH with a concentration <10 mg/mL and stored at r.t. (23 ± 1 °C).

For the synthesis of SiO_2 NH_2-50 and SiO_2 NH_2-100 custom-tailored colloidal, amorphous SiO_2 NPs were purchased from PlasmaChem GmbH (Germany) with a size of 50 nm and 90 nm. The particles were dispersed in EtOH with a concentration of 5 mg/mL and aminated using an excess of APTES under ambient conditions and a protecting gas atmosphere (Scheme S1 b)). A similar approach was chosen for the amination of Klebosol (30R50), a bimodal silica NP with a mean particle size of 80 nm,[3] purchased from Merck KGaA (Germany). The aminated particles were purified by dialysis (Nadir dialysis tube, MWCO approx. 10,000 - 20,000 Da, Carl Roth GmbH, Germany) for several days against double distilled water. The water was changed several times, leading to a very low ionic strength of the resulting particles. In a final step, the particles were centrifuged (15,000 rcf, 30 min) and redispersed in EtOH (SiO_2 NH_2-50 and SiO_2 NH_2-100) or in an acetate buffer (pH 3.5, Klebosol-NH_2) with particle concentrations <10 mg/mL.

2. Particle Characterization

2.1 Dynamic Light Scattering

Dynamic light scattering (DLS) measurements were performed on a Zetasizer Nano ZS from Malvern Panalytical (Germany). The instrument was equipped with a 4 mW He-Ne laser light source emitting at a wavelength of 633 nm. The scattered light was collected by an avalanche photodiode detector which was placed at a backscattering angle of 173°. Measurements were performed applying the backscattered mode in the Zetasizer Software (Version 8.02) to determine the z-average, polydispersity index (PDI), the intensity-based hydrodynamic diameter ($d_{h,i}$) and the number-based hydrodynamic diameter ($d_{h,0}$). For sample loading (about 1 mL), disposable acrylic cuvettes (REF 67.755, Sarstedt, Germany) were used. All DLS measurements were performed at an equilibrated temperature of 25 °C. The samples were measured in triplicate with each sample cuvette run with three measurements under

repeatability conditions. The raw data was analyzed by the standardized cumulants method (ISO 22412),[4] as provided by the software with a refractive index of 1.4649 for silica, a refractive index of 1.3300 and a viscosity of 0.8872 cP for water, or a refractive index of 1.361 and a viscosity of 1.0400 cP for EtOH.

In order, to guarantee the validity of the DLS results, each measurement sequence was started with performance qualification (PQ) measurements. These PQ measurements were performed with a dispersion of a particle size standard, nominal 100 nm polystyrene latex particles (PS-ST-B1261) from microparticles GmbH (Germany).

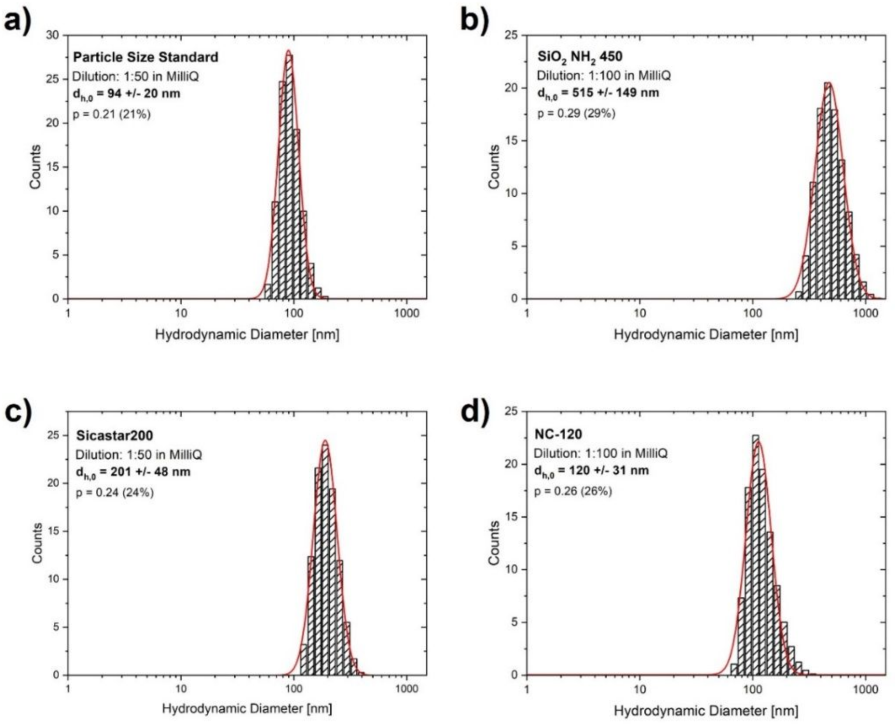

Figure S1. DLS results of the measured particle size standard for PQ measurements a), and the commercially available aminated SiO_2 NPs with sizes >100 nm (b) - d)).

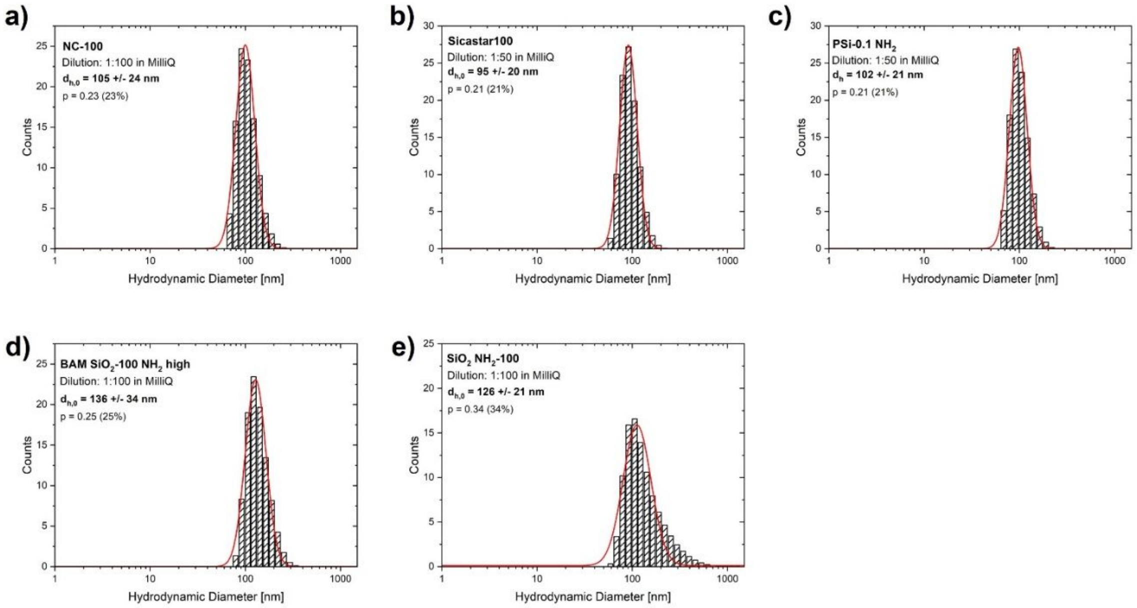

Figure S2. DLS results of the custom-made and commercially available aminated SiO_2 NPs with a nominal size of around 100 nm.

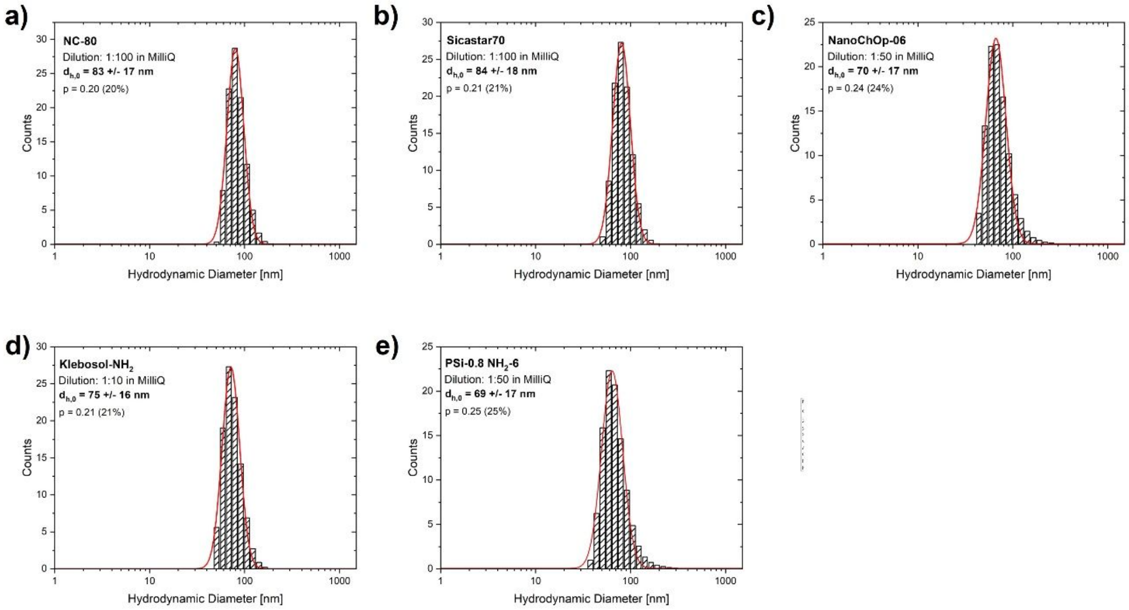

Figure S3. Number-based hydrodynamic diameters obtained by DLS measurements for the custom-made and commercially available aminated SiO_2 NPs with a nominal size of around 75 nm.

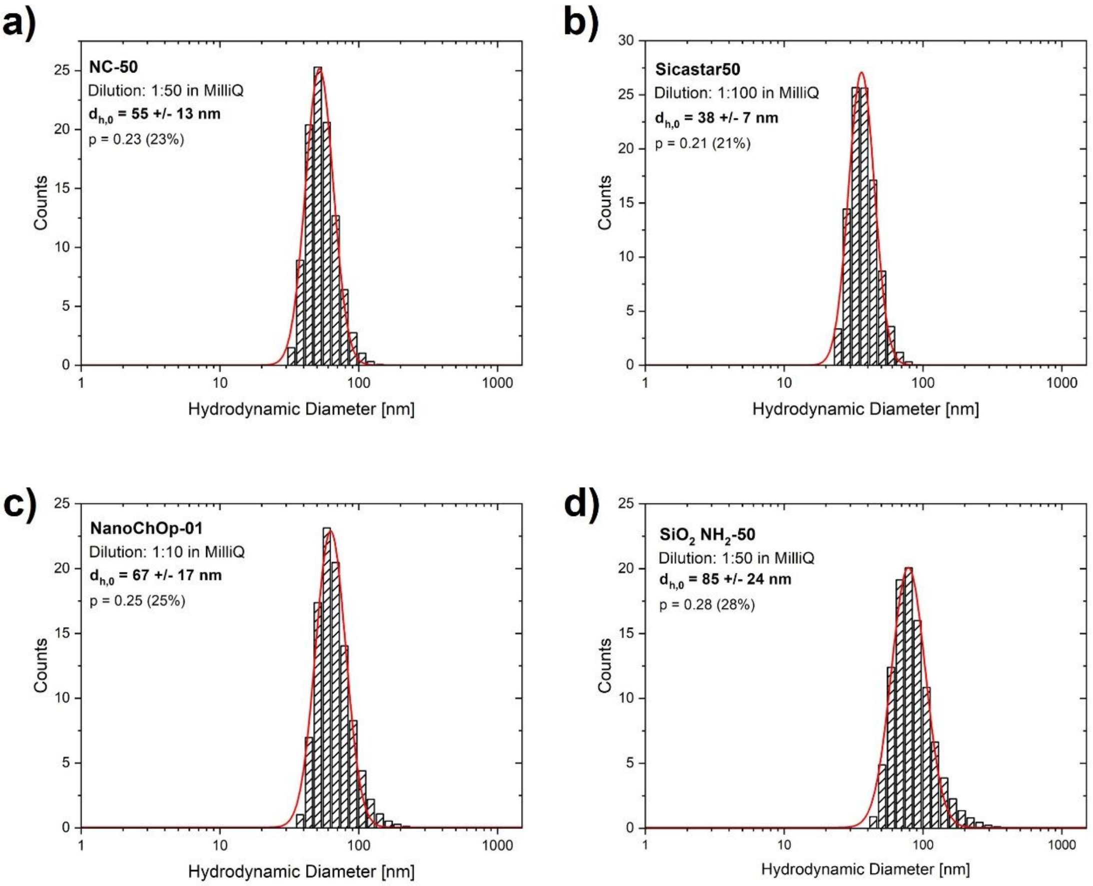

Figure S4. Overview of the measured number-based hydrodynamic diameters for the custom-made and commercially available aminated SiO_2 NPs with a nominal size of around 50 nm.

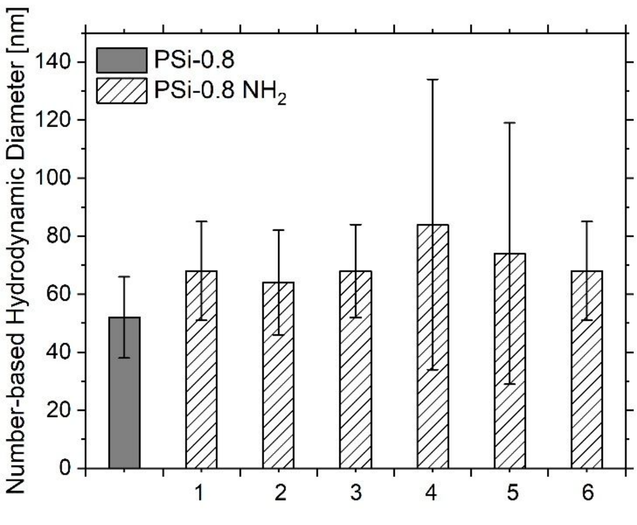

Figure S5. Overview of the number-based hydrodynamic diameters prior (PSi-0.8, grey) and after the grafting with different amounts of APTES (Psi-0.8 NH_2); 1 = 1 eq. APTES; 2 = 2.5 eq. APTES; 3 = 5 eq. APTES; 4 = 7.5 eq. APTES; 5 = 10 eq. APTES; 6 = 25 eq. APTES compared to the calculated amount for a monolayer.

2.2 Nanoparticle Tracking Analysis (NTA)

DLS is relatively easy to use and requires minimal sample preparation. Also, it provides rapid measurements for a wide range of particle sizes from nanometer to micrometer scale. Therefore, it is suitable for high-throughput applications. Nevertheless, it is limited for analyzing of more polydisperse samples due to a lower resolution for samples with a broad size distribution and higher sensitivity to larger particles. Compared to this NTA provides a higher resolution size distribution profile, allowing for detailed analysis of heterogeneous samples and can measure particle concentration accurately, which is beneficial for quantifying samples. Even NTA is more time-consuming than DLS, it is also well suited for real-time measurements. DLS and NTA are both non-destructive NM characterization methods, widely used in research and industry, and are usable as online, in-line, and at-line monitoring in automated workflows. Due to the size limitations of the NTA technique for SiO_2 NPs, only aqueous dispersions of SiO_2 NPs (>75 nm) were characterized by NTA to validate $d_{h,0}$ and the particle number concentration (PNC). The utilized NanoSight LM 10 system from Malvern Panalytical (Germany) was equipped with a 405 nm laser and the measurements were performed at a temperature of 25 ˚C in static mode, following the standards ISO19430 and ASTM E2834.[5, 6] The NanoSight NTA software (Version: 3.32) was used to capture 5 videos with 60 s and 25 fps of the scattering of the highly diluted samples. A comparison of the obtained results from DLS (grey) and NTA (white with crossed lines) are given in Figure S6.

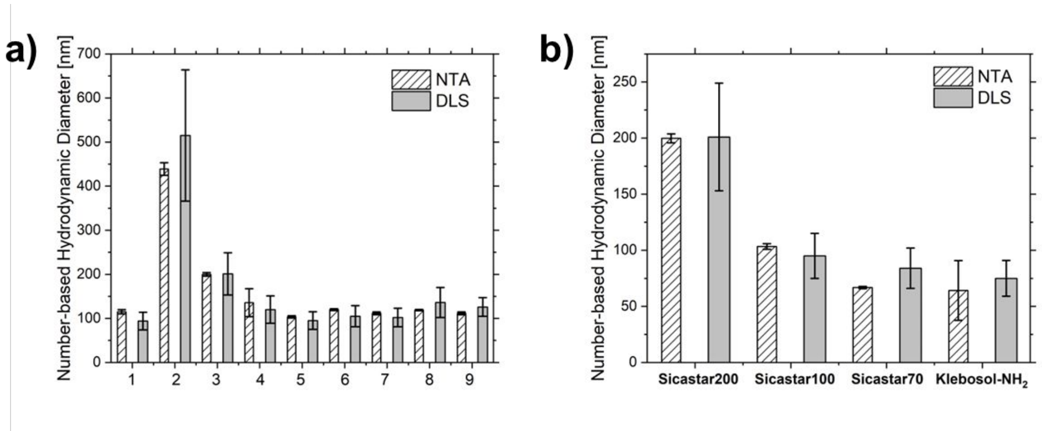

Figure S6. Overview of the number-based hydrodynamic diameters obtained by DLS (grey) and NTA (white with crossed lines) measurements for the aminated SiO_2 NPs with a nominal size >75 nm: a) 1 = particle size standard; 2 = micro450; 3 = sicastar200; 4 = NC-120; 5 = sicastar100; 6 = NC-100; 7= PSi-0.1 NH_2; 8 = BAM SiO_2-

100 NH_2 high; 9 = SiO_2 NH_2-100. b) Results of the DLS and NTA comparison for the different sized Sicastar particles and Klebosol-NH_2.

2.3 Zeta Potential Measurements

Zeta potential measurements were performed on a Zetasizer Nano ZS from Malvern Panalytical (Germany). The instrument was equipped with a 4 mW He-Ne laser light source emitting at a wavelength of 633 nm. The scattered light was collected by an avalanche photodiode detector which is placed at a forward scattering angle of 13°. Prior to sample loading, disposable polycarbonate folded capillary cells (DTS-1670, Malvern Panalytical) with gold plated beryllium-copper electrodes were pre-rinsed with analytical grade EtOH and excessively rinsed with MilliQ-water. The sample cells were filled with 0.8 – 1.0 mL of the diluted sample dispersion. Samples were diluted 1:100 or 1:50 in MilliQ-water or EtOH. All measurements were performed at an equilibrated temperature of 25 °C. Each sample cell was run in triplicate with three 3 runs per measurement under repeatability conditions. The raw data was analyzed by applying the Smoluchowski model, assuming a refractive index of 1.4649 for silica, a refractive index of 1.3300 and a viscosity of 0.8872 cP for water, or a refractive index of 1.361 and a viscosity of 1.0400 cP for EtOH. Prior to the sample measurements the accuracy of the device was tested with a NIST-traceable particle size standard based on polystyrene nanoparticles (100 nm, PS-ST-B1261) from microparticles GmbH (Germany).

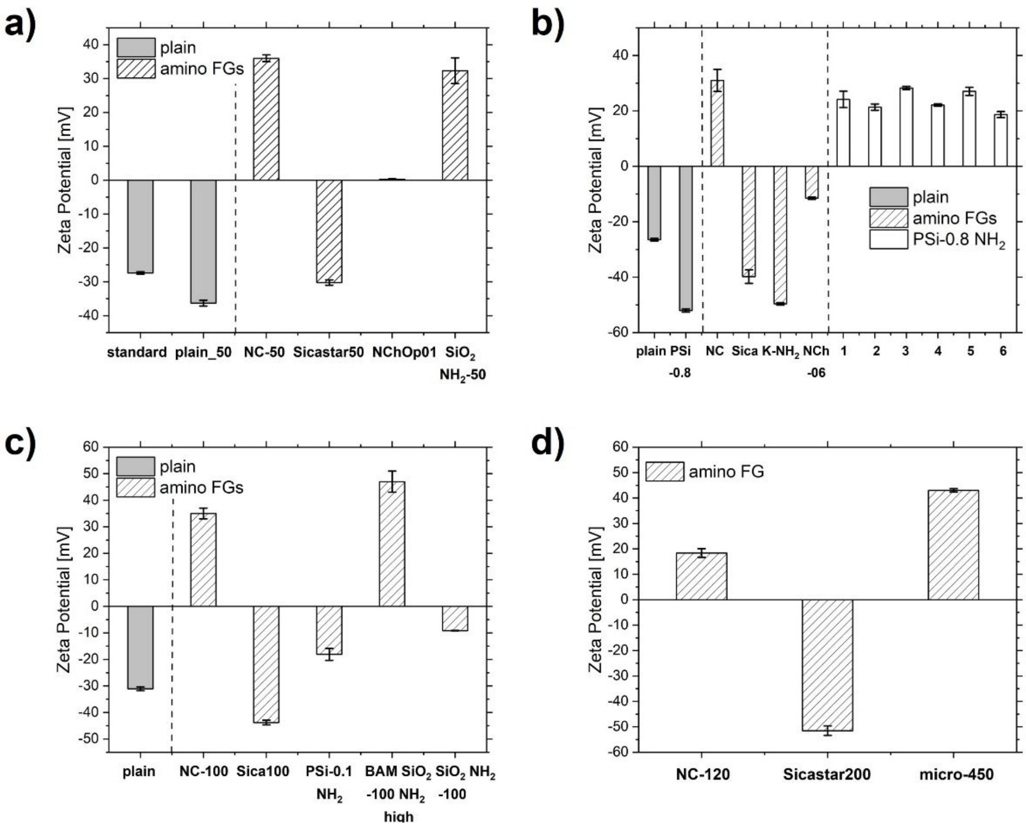

Figure S7. Overview of the zeta potential measurements for 50 nm (a), 75 nm (b), 100 nm (c), and >100 nm (d) sized aminated SiO_2 NPs.

2.4 Estimation of a monolayer of amino FGs

The specific surface area (SSA) of the aminated SiO_2 NPs was estimated from particle diameters obtained by DLS or NTA measurements, using a silica density of 2.20 g/cm^3 for the particles purchased from Nanocomposix (NC-size) and 2.00 g/cm^3 for the other particles. The SSA was calculated with in equation (1):

$$\text{Specific Surface Area: SSA [m}^2\text{/g]} = A / m = (4/3 \cdot \pi \cdot r^2) / (d \cdot 4/3 \cdot \pi \cdot r^3) = 6 / d \tag{1}$$

Table S1. Overview of the results obtained from the particle characterization and estimation of the amount of amino groups/Fluram per monolayer for the aminated SiO_2 NPs with sizes of around 50 nm and 75 nm used in this study.

Particle Name	$d_{h,0}$ (DLS) [nm]	Zeta potential [mV]	SSA [m^2/g]	Estimated amount of amino FGs for one monolayer [µmol/g]	Estimated Fluram per monolayer [µmol/g]
NC-50	55 ± 13	36.0 ± 1.0	49.59	329	54
NChOp-01	67 ± 17	0.3 ± 0.1	40.71	270	45
Sicastar 50	38 ± 7	-30.2 ± 0.8	143.54	477	157
SiO_2-NH_2-50	85 ± 24	32.3 ± 3.8	64.17	213	70
NC-80	83 ± 17	31.0 ± 4.0	32.86	218	36
Sicastar 70	84 ± 14	-39.8 ± 2.4	32.47	216	36
Klebosol-NH_2	75 ± 16	-49.6 ± 0.4	36.36	242	40
NChOp-06	70 ± 17	-11.5 ± 0.4	38.96	259	43

Table S2. Overview of the results obtained from the particle characterization and estimation of the amount of amino groups/Fluram per monolayer for the aminated SiO_2 NPs with sizes of ≥100 nm; n.d. = not determined.

Particle Name	$d_{h,0}$ (DLS) [nm]	$d_{h,0}$ (NTA) [nm]	PNC (NTA) [particles/ mL]	Zeta potential [mV]	SSA [m^2/g]	Estimated amount of amino FGs for one monolayer [µmol/g]	Estimated Fluram per monolayer [µmol/g]
NC-100	105 ± 24	120 ± 2	6E12 ± 1E11	35.0 ± 2.0	25.50	173	28
Sicastar 100	95 ± 20	103 ± 3	2E8 ± 3E7	-43.8 ± 0.9	28.71	191	31
PSi-0.1 NH_2	102 ± 21	112 ± 3	1E8 ± 1E7	-18.1 ± 2.3	26.74	178	29
BAM SiO_2-100 NH_2 high	136 ± 24	119 ± 2	2E9 ± 2E8	47.0 ± 4.0	20.05	133	22
SiO_2-NH_2 100	126 ± 21	111 ± 4	n.d.	-9.1 ± 0.1	21.65	144	24
NC-120	120 ± 31	136 ± 32	2E7 ± 6E6	18.4 ± 1.7	22.73	151	25
Sicastar 200	201 ± 48	200 ± 4	2E9 ± 2E8	-51.5 ± 1.8	13.57	90	15
micro450	515 ± 149	439 ± 14	1E9± 1E8	43.0 ± 0.7	6.21	41	7

Table S3. Overview of the results obtained from the particle characterization and estimation of the amount of amino groups/Fluram per monolayer for the plain and aminated PSi-0.8 with different used concentrations of APTES during the grafting step; n.d. = not determined.

Particle Name	$d_{h,0}$ (DLS) [nm]	Zeta potential [mV]	SSA [m^2/g]	Estimated amount of amino FGs for one monolayer [µmol/g]	Estimated Fluram per monolayer [µmol/g]
plain PSi-0.8	52 ± 14	-52 ± 1	52.45	n.d.	n.d.
PSi-0.8 NH_2-1	68 ± 17	36 ± 1	40.11	267	44
PSi-0.8 NH_2-2	64 ± 18	24 ± 3	42.61	283	47
PSi-0.8 NH_2-3	68 ± 16	35 ± 1	40.11	267	44
PSi-0.8 NH_2-4	84 ± 50	24 ± 2	32.47	216	36
PSi-0.8 NH_2-5	74 ± 45	27 ± 1	36.86	245	40
PSi-0.8 NH_2-6	68 ± 17	19 ± 2	40.11	267	44

3. Quantification of the Total Amount of Amino Functional Groups using a pH Titration Approach

Quantifying the total amount of amino functional groups on silica nanoparticles using an optical or potentiometric pH titration approach involves determining the protonation states of these groups at various pH levels. Amino groups on the surface of silica nanoparticles can accept protons, transitioning from $–NH_2$ to $–NH_3^+$. By titrating the nanoparticle suspension with a strong acid or base and monitoring the pH changes, not only the pK_a values of the amino groups can be identified, but also the titration curve of a back titration approach provides insights into the total amount of amino functional groups present on the nanoparticles.

Starting with literature-known approaches,[7-10] we adapted and optimized them to prevent any dissolution of the particles by choosing concentrations of the NaOH and HCl solutions to work in a pH range of 3 – 9. Therefore, we calculated the consumption of HCl for a theoretically monolayer of the particles, assuming 4 APTES molecules per nm^2. This resulted in a range of 363 – 776 nmol/mg APTES for the different particle sizes and a consumption of 0.036 - 0.076 mL of HCl (0.1 M) or 0.36 – 0.76 mL of HCl (0.01 M) per mg particles. For better handling with our classical 10 mL glass burettes (DIN AS ± 0.02 mL), we used a volume

of 5 mL of HCl with a concentration of 0.001 M and 3 - 5 mg of particles. All starting points of the different titration were >pH 3.5 exemplarily shown in Figure S8a). Although principally, a potentiometric pH titration enables to distinguish between different (de)protonable FGs varying in pK_a, we decided to use a back titration approach for our particles, in which changes in the point of zero charge of different ionic groups cannot be exploited anymore for a discrimination, but for different concentrations of one type of FG (Figure S8 b).

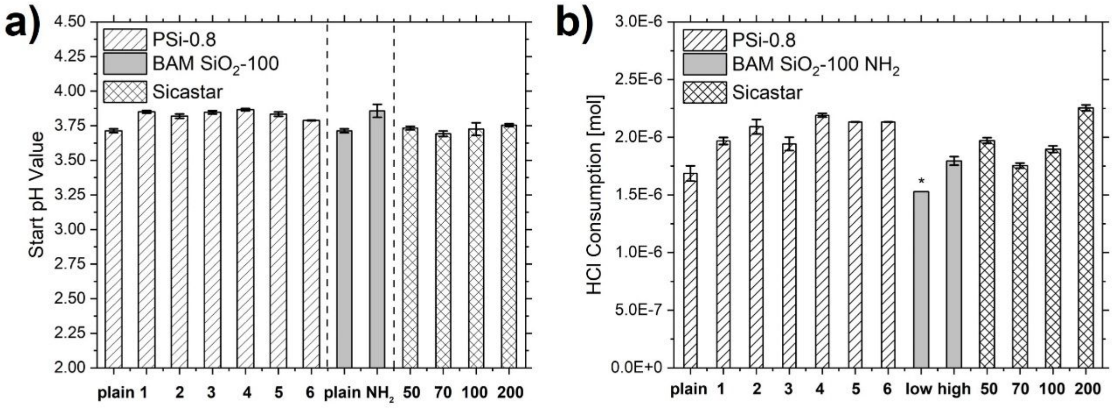

Figure S8. Starting points of the pH measurements after incubation for plain and differently aminated SiO_2 NPs (a) and the associated consumption of HCl (b).

3.1 Determination of the titrants

All experiments to determine the total amount of amino functional groups using an acid-base pH titration were performed with freshly prepared 0.001 M HCl and 0.001 M NaOH on a weekly basis. These were prepared from commercially available 0.01 M NaOH/HCl solutions (Carl Roth GmbH, Germany) by measuring 50 mL of the 0.01 M solution gravimetrically or with a volumetric pipette (AS ± 0.05 mL, Brand GmbH, Germany), transferring and rinsing with MilliQ-water into a volumetric flask until the total volume was reached, followed by mixing of the solution to ensure uniform concentration. The titer of the prepared 1 mM HCl and 1 mM NaOH solutions utilized for the different pH titrations was determined using 0.01 M volumetric standard solution of HCl/NaOH (Carl Roth GmbH, Germany) with known titer (20 °C) of 0.9980 - 1.020 (NaOH) and 0.9950 – 1.0050 (HCl), respectively. 0.5 mL of the volumetric standards were mixed with 20 mL of MilliQ-water at room temperature (r.t. = 23 ± 1 °C). The mixture was stirred, and the initial pH value was measured using a

SevenExcellence S475 pH meter (Mettler Toledo, Germany) and a pH electrode (InLab® Micro-Pro-ISM, Mettler Toledo, Germany). The titrants were then gradually added until a pH of 7.0 was reached. The titer for NaOH and HCl were calculated using equation (2) and (3):

$$t_{HCl\ 1\ mM} = \frac{c_{NaOH} * V_{NaOH} * t_{NaOH}}{c_{HCl} * V_{HCl}} = \frac{0.01\ mol/L * 0.0005\ L * 1.000}{\frac{0.001\ mol}{L} * 0.00434L} = 1.15207 \quad (2)$$

$$t_{NaOH\ 1\ mM} = \frac{c_{HCl} * V_{HCl} * t_{HCl}}{c_{NaOH} * V_{NaOH}} = \frac{0.01\ mol/L * 0.0005\ L * 1.000}{\frac{0.001\ mol}{L} * 0.00522L} = 0.95785 \quad (3)$$

3.2 Optical and potentiometric pH -Titration

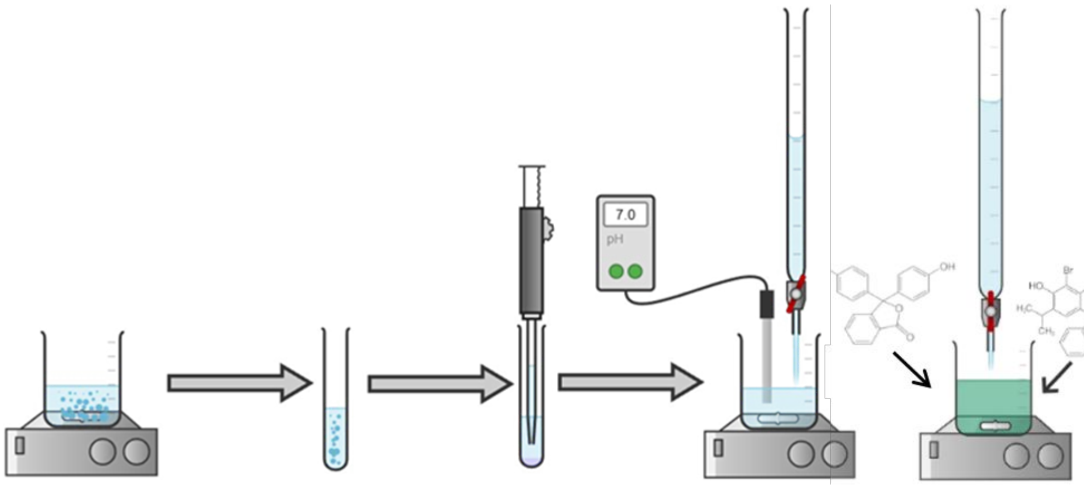

Figure S9. Scheme of the optical and potentiometric pH titration without particles. After incubation of the dried particles with known concentration in 1 mM HCl for 1 – 3 h at r.t., the particle dispersion was centrifuged, and the supernatant was separated. Next, a known volume of the supernatant was titrated against 1 mM NaOH using a potentiometric determination (pH = 7.0) or optical determination.

For both measurement approaches an optimized workflow was developed, involving a purification and drying step. Centrifugation tubes (2 mL safe Lock, Eppendorf GmbH, Germany) were dried overnight at 80 ˚C and weighed after colling (balance Cubis MCM (Sartorius, Germany)) prior purification of the samples by centrifugation of the particle dispersions for 30 min at r.t. (21,000 rcf, Eppendorf 5424R, Eppendorf GmbH, Germany). Next the supernatant was removed, and the precipitated particles were dried overnight. The dried particles were weighed, redispersed in 1 mL of MilliQ-water (MilliQ-water, 0.055 µS m^{-1};

Merck Milli-Q® IQ 700 device, stored for at least one day), using a calibrated Eppendorf pipette (systematic error 0.3% - 2.5%), and mixed with 5 mL of a 0.001 M HCl solution (Eppendorf pipette 5 mL, systematic error 0.6% - 3.0%). This mixture was then incubated for at least 1 h to 3 h under gentle stirring at r.t. to assure complete protonation of the surface amino FGs. After the incubation the particle dispersion was centrifuged (15,000 rcf, 30 min) and 5 mL of the supernatant was transferred gravimetrically or with a volumetric pipette into a flask with 20 mL of MilliQ-water. Finally, the sample was titrated with a 0.001 M NaOH solution, using a 10 mL burette (AS, ± 0.02 mL, Brand, Germany). Thereby, the amount of 0.001 M NaOH solution required to reach the equivalence point of the acid-base-titration of pH 7 was determined with a calibrated pH-meter (Seven Excellence S475 pH meter, Mettler Toledo, Germany) and a very sensitive pH electrode (InLab® Micro-Pro-ISM, Mettler Toledo, Germany). The volume of used NaOH was utilized to calculate the amount of consumed HCl and the amount of surface amino FGs on the aminated SiO_2 NP. Data evaluation was done with a custom-made software. All measurements were typically done in triplicate at a constant temperature of (23 ± 1) °C.
The optical determination via this back titration approach was performed similar with the gravimetrically addition of a known amount of BTB indicator solution prior titration. The endpoint of the titration was reached with the color change from yellow to green.

3.3 Determination of the Amount of amino FGs

For our titration approach we assumed that the titration reaction was completed and that the stoichiometry of the reaction is 1:1 (one mole of NaOH reacts with one mole of HCl). In the following we show the quantification of the total number of amino FGs exemplary with PSi-0.1 NH_2:

$$HCl(aq) + NaOH(aq) \rightarrow NaCl(aq) + H_2O(l)$$

4.82 mL of NaOH were used during the titration to reach a pH of 7.00, resulting in 4.61 µmol of NaOH reacted with the excess of HCl in the supernatant (4).

$n(HCl_{excess}) = n(NaOH) = c(NaOH(aq)) * V(NaOH(aq)) * t(NaOH\ 1mM)$ (4)

$= 0.001\ mol/L * 0.00482\ L * 0.95785 = 4.61\ \mu mol$

This was used to calculate the amount of HCl that reacted with the amino FGs on the particle surface (5).

$n(HCl_{reacted}\ \text{with amino FGs}) = n(HCl_{total\ addition}) - n(HCl_{excess})$ (5)

$= (5.004\ \mu mol*1.15) - 4.61\ \mu mol = 1.14\ \mu mol$

5 mg of particles were used in total for this experiment, therefore 229 nmol of amino FGs are in 1 mg of particles.

The amount of amino FGs per nm^2 was estimated from the average hydrodynamic diameter and a silica density of 2.0 g/cm^3 using the equations (6) - (11):

Surface area of a particle $[nm^2] = 4 * \pi * r^2 = 4 * \pi * (50\ nm)^2 = 31416\ nm^2$ (6)

Voulme of a particle $[m^3] = V = 4/3*\pi * r^3 = 4/3 * \pi * (50*10^{-9}\ m)^3 = 5.22\cdot10^{-22}\ m^3$ (7)

Mass of a particle $[g] = \rho(SiO_2) * V = 2000000\ g/m^3 * 5.22\cdot10^{-22}\ m^3 = 1.05\cdot10^{-15}\ g$ (8)

Number of particles per mg $= 0.001\ g/\ 1.05\cdot10^{-15}\ g = 9.58\cdot10^{11}$ (9)

Amount of amino FGs per particle $[mol] = 0.229\cdot10^{-6}\ mol/9.58\cdot10^{11} = 2.39\cdot10^{-19}\ mol$ (10)

Number of amino FGs per $nm^2 = (2.39\cdot10^{-19}\ mol*6.022\cdot10^{23}\ 1/mol)/10243\ nm^2 = 4.58$ (11)

Note: Several assumptions and simplifications were used to estimate the volume, mass and surface area of one particle. We assumed perfect, monodisperse spheres with a bulk density of 2.0 g/cm^3 for silica and a molar mass of $M(SiO_2) = 60.1$ g/mol. For the calculation of the density of amino FGs/nm^2 and the coverage of a monolayer, we assumed a maximum number of 4 APTES molecules per nm^2.[2]

3.4 Bilateral comparison BAM and PolyAn

To explore the reliability and robustness of our workflow for manually performed titration, we studied the influence of the operator and the laboratory equipment (Table S4) in a bilateral comparison with a SME, PolyAn. We tested the potential influence of the utilized electrode and sample preparation steps, i.e., purification steps, drying, and weighing of the particles prior to their incubation with 0.001 M HCl.

Table S4. Overview of the used equipment in the different laboratories for the bilateral comparison.

Equipment	BAM	PolyAn
Centrifuge	Hettich Rotina 380R, 16,000 rcf (30 min)<br>Eppendorf 5240R, 21,000 rcf (30 min)	Sigma 1-14, 16,160 rcf (30 min)<br>Hettich Rotina 380 R, 21,000 rcf (30 min)
Balance	Satorius Cubis MCM	Kern, ABS 120-4N
Pipettes	Eppendorf Research Plus pipettes (single channel, variable) 100 – 1000 µL (blue), 250 - 2500 µL (red), 1000 – 5000 µL (violet), 1000 -10000 µL (green) with the recommended epT.I.P.S. (Eppendorf)	Socorex: Acura 825<br>(100 – 1000 µL)
Centrifuge Tubes	1.5 mL or 2.0 mL SafeLock (Eppendorf, Germany)	1.7 mL Safeseal (Sorenson BioSience, US)
pH-meter	Mettler Toledo Seven Excellence S475	Mettler Toledo: Five Easy
pH electrode	InLab® Micro-Pro-ISM	LE410 Mettler Toledo
Glass burette	10 mL burette (AS, ±.0.02 mL)	10 mL, (AS, ± 0.03 mL)

3.5 Potential Influences of the sample Preparation on the Potentiometric Back Titration

Sample preparation plays a crucial role in our potentiometric back titration approach, that might influence the accuracy and reliability of the results. Therefore, we tested the drying step of the sample (Figure S11) to ensure that no moisture interfere with the particle weighing and titration process, which could otherwise lead to erroneous readings. We also tested the purification of the sample by separating the supernatant from the precipitate (Figure S10). In addition, precise pipetting is important to ensure that the correct concentration of particles is used, as even minor deviations can significantly impact the outcome. For reproducibility and reliability, each step in the sample preparation must be carefully controlled to ensure the integrity of the potentiometric back titration.

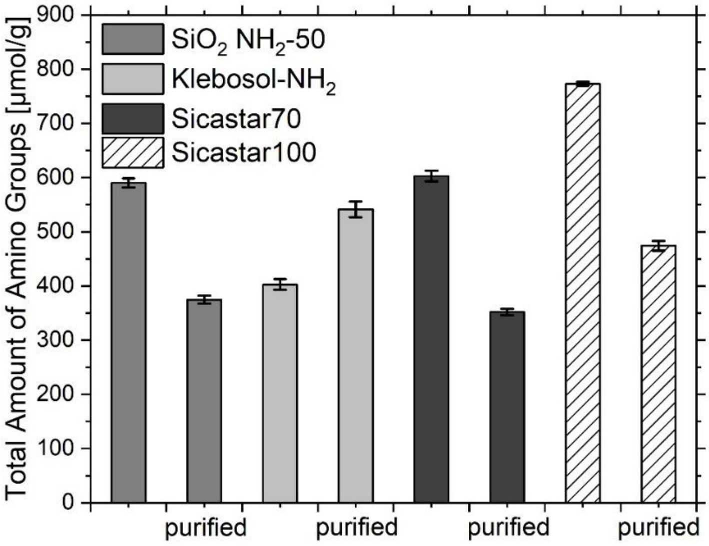

Figure S10. Influence of the centrifugation step to separate free amino groups from the SiO_2 NPs prior the drying process. Klebosol-NH_2 was stored in acetate buffer (pH 3.5), while SiO_2 NH_2-50, Sicastar70, and Sicastar100 were stored in EtOH.

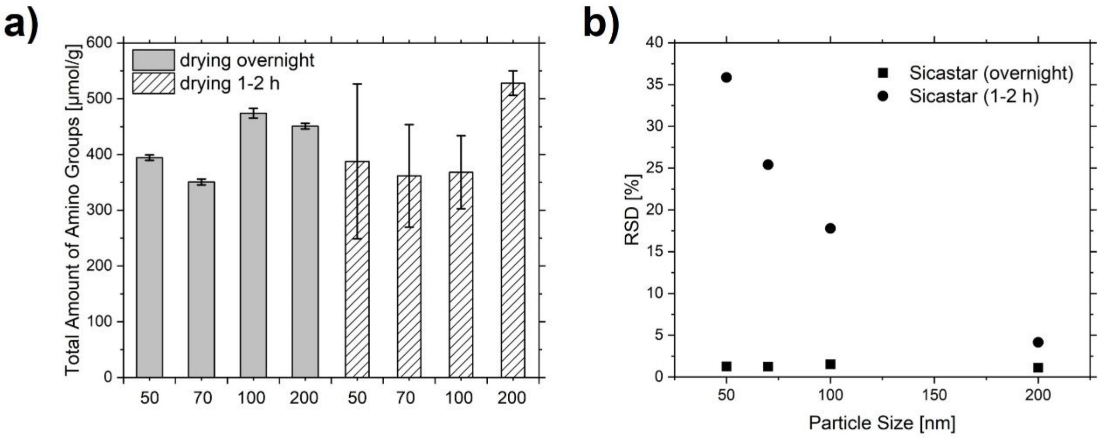

Figure S11. Influence of the drying process exemplarily shown for the Sicastar particles of different sizes. The particles were dried in one laboratory overnight, resulting in constant RSD values of 1.1-1.5%, while in a second laboratory the particles were dried briefly for 1-2 h, resulting in size-dependent RSD values of 35.9% (Sicastar50) to 4.2% (Sicastar200).

4. Validation with TGA and qNMR

For cross-validation of the obtained results by our potentiometric back titration, we performed measurement with TGA and qNMR.

4.1 TGA

For the TGA measurements performed with a calibrated NEXTA Series Simultaneous Thermogravimetric Analyzer (STA200RV, Hitachi) equipped with an autosampler, the dispersed particles were removed from the dispersion by centrifugation for 30 min (centrifuge 5424R, Eppendorf; 21,000 rcf) and dried overnight in an oven at 80 ˚C. The dried particles were weighed with the microbalance Cubis MSM6.7 (Sartorius, Germany) using an amount of SiO_2 NP in the range of 3 mg to 4 mg. Each sample was measured in triplicate.

The following measurement procedure was applied for all samples: 1) 60 min at 30 ˚C to stabilize the initial sample mass; 2) 60 min at 100 ˚C to remove water adsorbed at the surface; 3) heating from 100 ˚C to 1000 ˚C with a heat rate of 10 ˚C/min. All steps were performed under Argon flow (200 mL/min) as protective gas. An overview of the measurement procedure is depicted in Fig. S12 a) as a dashed red line. Data evaluation was performed by determining the weight loss during step 3 for the range 100 ˚C to 650 ˚C as a mass instability at T >650 ˚C was observed which is most probably attributed to sample instability. Due to lack of suitable blank sample, the entire wight loss was attributed to APTES-fragments loss during the sample treatment. For calculation the total amount of APTES (n in nmol) a molecular weight M = 69.0861 g/mol was assumed according to the literature.[11, 12] The total amount of APTES (and thus amino groups) per sample mass (in nmol/mg) was calculated using the sample mass at 100 ˚C (at the beginning of step 3). The obtained results are summarized in Table S5. The obtained TG graphs for all samples are depicted in Fig. S12.

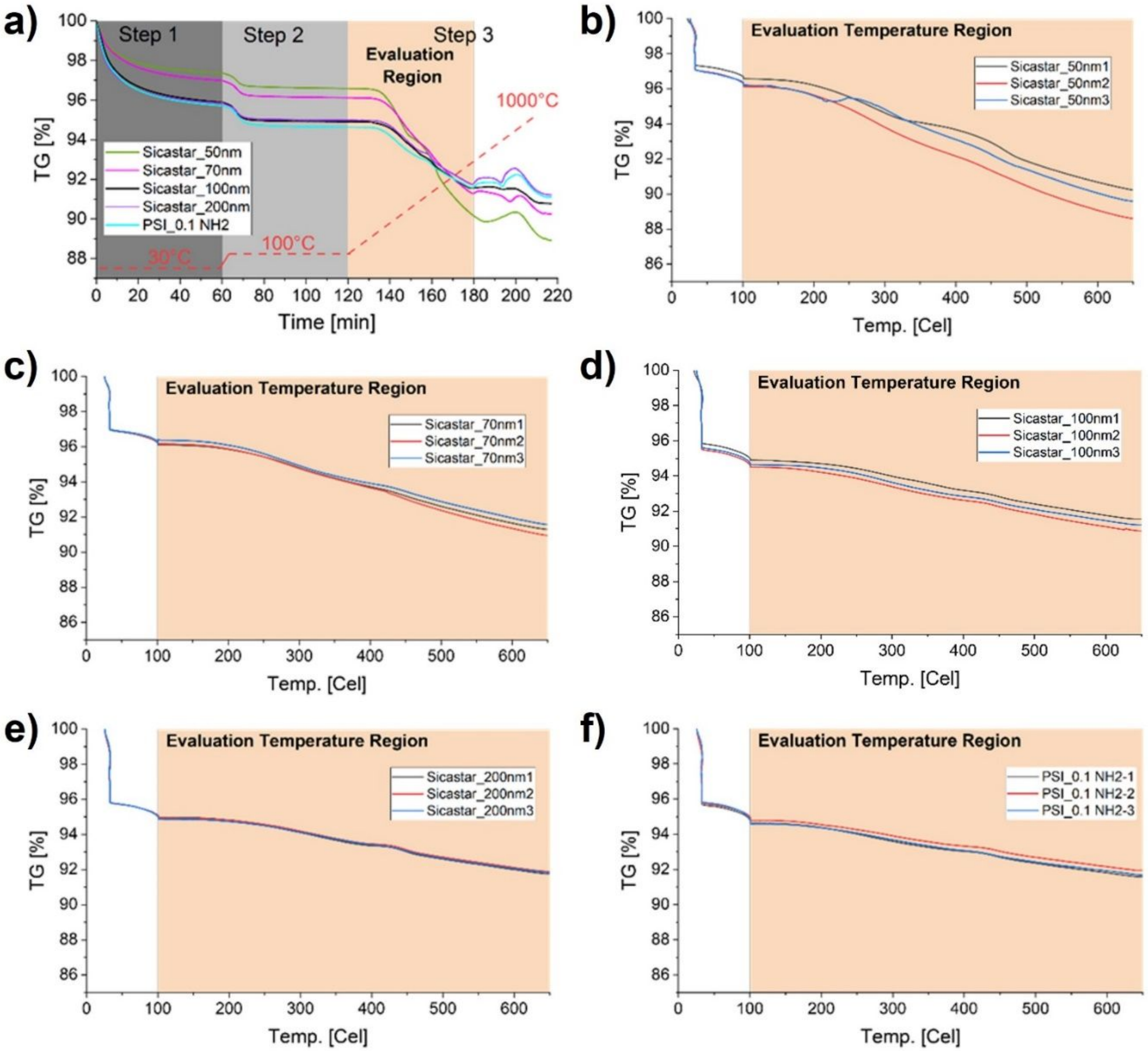

Figure S12. a) Overview of the measurement procedure with step 1 (mass stabilization, 60 min at 30 ˚C), step 2 (sample drying, 60 min at 100 ˚C) and step 3 (heating from 100 ˚C to 1000 ˚C with a heating rate of 10 ˚C/min), with highlighted evaluation region (100 ˚C – 650 ˚C). An exemplary TG graph for each analyzed sample is depict as function of time illustrating that the sample mass was stabilized prior the evaluation region. b) - f) TG graphs in triplicate for the analyzed samples Sicastar_50, Sicastar_70, Sicastar_100, Sicastar_200 and PSI-0.1 NH_2.

Table S5. Calculated amino groups per mg particle for the studied samples.

Sample	Amount of determined amino FGs [µmol/mg]
**Sicastar 50**	1027 ± 70
**Sicastar 70**	746 ± 26
**Sicastar 100**	530 ± 18
**Sicastar 200**	474 ± 12
**PSI-0.1 NH_2**	449 ± 11

4.2 qNMR

The total amount of amino FGs was determined by qNMR, employing an adapted protocol from our previous work.[13] Therefore, the aminated SiO_2 NPs were removed from aqueous or ethanolic dispersion by centrifugation (21,000 rcf, 30 min), dried in centrifuge tubes (safe lock 1.5 mL or 2.0 mL, Eppendorf GmbH, Germany) at an elevated temperature (80 ˚C) overnight, weighed with an ultra-micro balance (Cubis MCM 6.7, Satorius, Germany), and dissolved by addition of a 1 M sodium deuteroxide solution (NaOD, Sigma Aldrich) in D_2O (Sigma Aldrich) at 50 ˚C at least for 4 h under gentle mixing. Since it does not display signals in the frequency window employed for amino FG quantification at 2.4 ppm and 0.3 ppm originating from the aliphatic CH_2 groups of the aminosilane grafted to the SiO_2 NP surface, ultrapure maleic acid (TraceCERT®, Sigma Aldrich) was added as an internal standard (2H, 6.3 ppm) to the sample solutions. NMR experiments were performed on a 600 MHz JEOL ECZ spectrometer, where a 90˚ pulse angle, a pulse delay of 50 s, 64 scans, an acquisition time of 3.6 s, and a spectral width of 30 ppm were used.

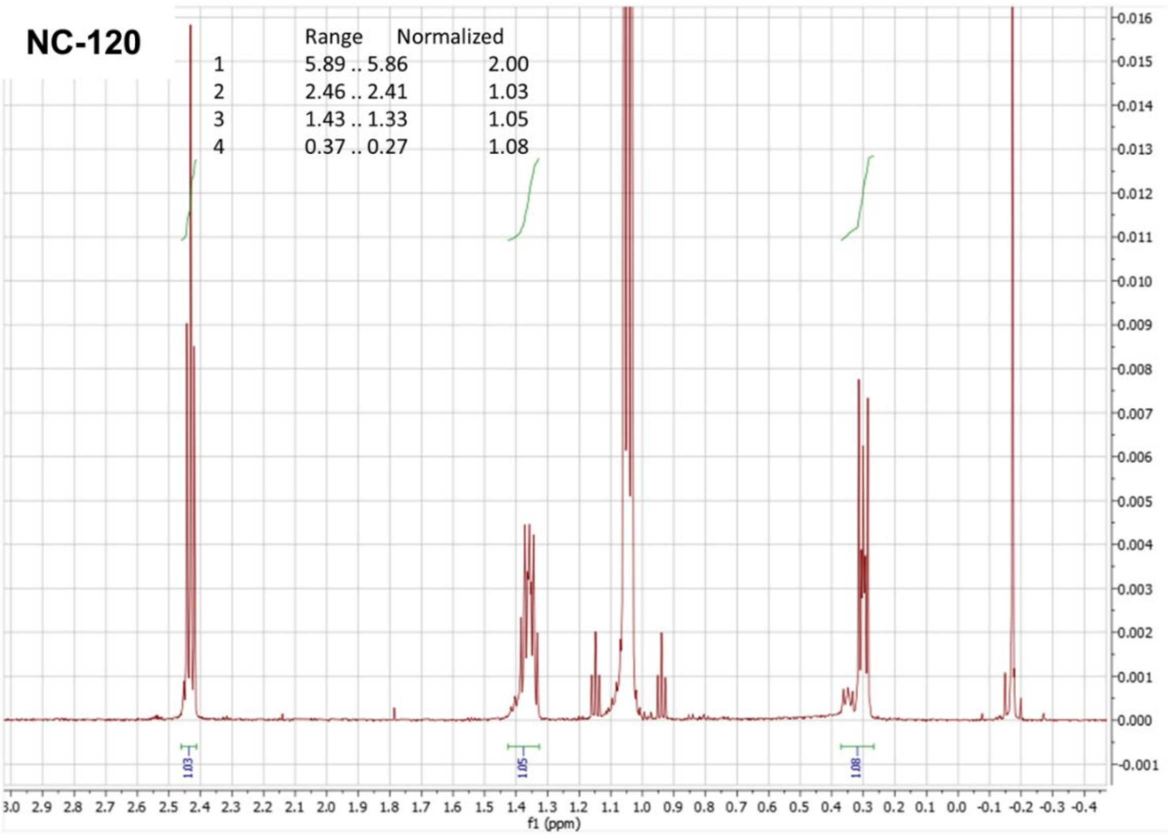

Figure S13. qNMR Spectrum of NC-120 in 1 M NaOD/D_2O. The peaks of the aminopropyl group (2.41-2.46 and 0.27-0.37) were used with an internal standard (maleic acid) to quantify the total amount of amino FGs.

5. Fluram Assay

The reporter-accessible amount of amino FGs was determined by an optical Fluram assay recently semi-automated by us using a two-step approach with a pipetting robot (Assist Plus, INTEGRA Bioscience, Switzerland).[2] Fluorescamine (Fluram, 1.51 µmol/15 µL, abcr GmbH, Germany) was dissolved in 954 µL of acetonitrile (Sigma Aldrich, Germany). Next 15 µL of it was added to the previous purified particle samples (0.2 - 0.5 mg), the negative and positive controls, and the samples of the calibration curve dispersed in 1 mL of phosphate buffer (0.01 M, pH 8), respectively. The mixtures were briefly homogenized, followed by incubation for 40 min under gentle shaking. After incubation, all samples were transferred to 96-well microtiter plates (µClear, Greiner Bio-One, Germany), using 3x 200 µL (3 wells) per sample. Assay readout was done with the Infinite M200 pro microplate reader from Tecan (Switzerland) using an excitation wavelength λ_{ex} of 392 nm and an emission wavelength λ_{em} of 480 nm. A calibration curve using ethanolamine (Sigma Aldrich, Germany) as a standard in phosphate

buffer (pH 8.3) with 12 different concentrations within the concentration range of 0.37 nmol – 44.7 nmol was prepared to quantify the amount of amino FGs on the NP samples. Also, the systematic error of the pipettes had to be considered for the performance of the assay. These systematic errors amounted to 6% (5 – 1250 µL) and 10% (0.5 – 300 µL), depending on the volume of the D-One single channel pipettes (INTEGRA Bioscience, Switzerland).

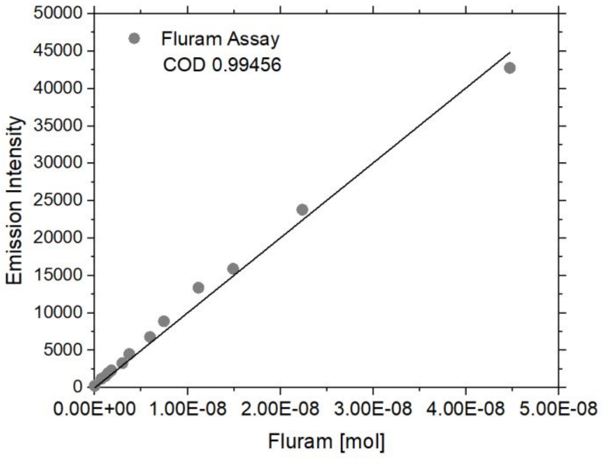

Figure S14. Calibration curve of the Fluram assay, using 12 different concentrations of ethanolamine in a range of 0.37 – 44.7 nmol.

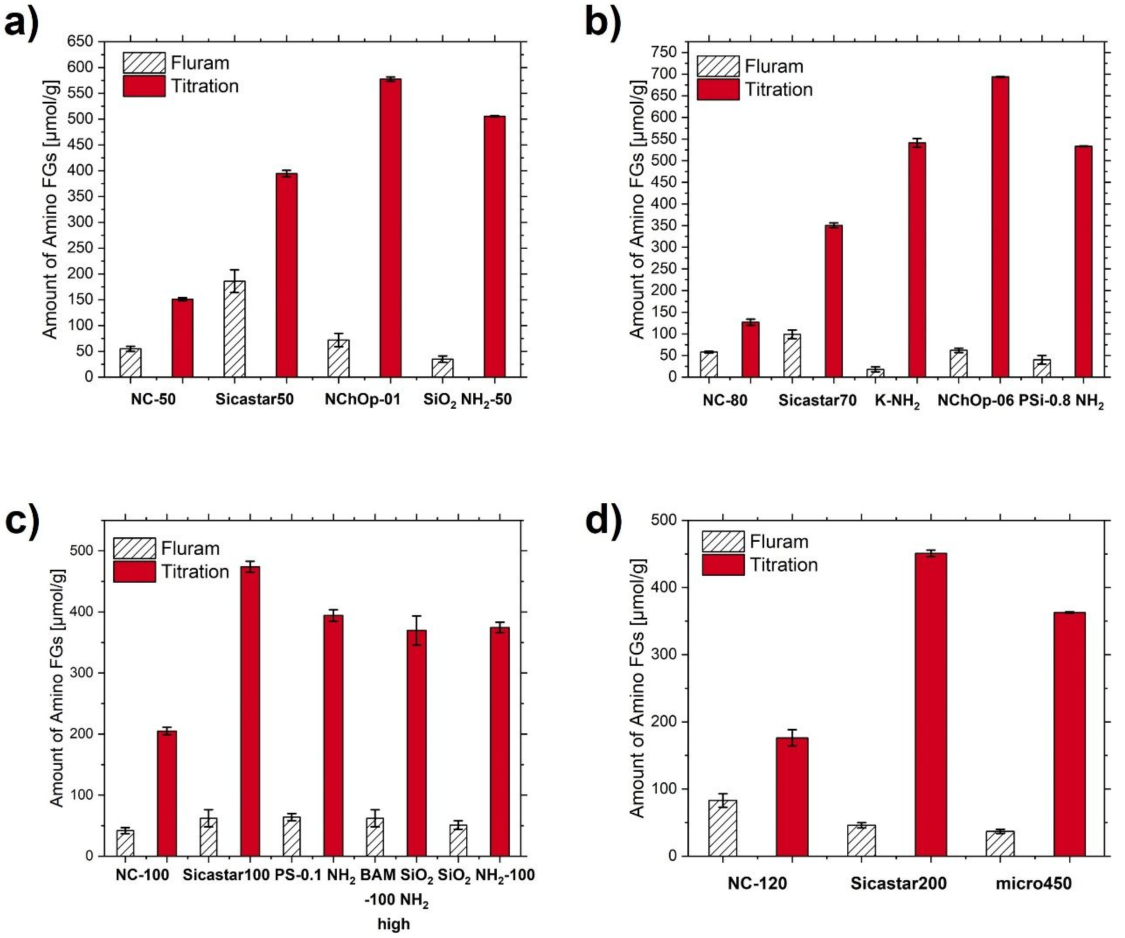

Figure S15. Comparison and correlation of the reporter-accessible amount (Fluram) and total amount (titration) of amino FGs for the set of 50 nm (a), 75 nm (b); 100 nm (c), and >100 nm sized aminated SiO_2 NPs.

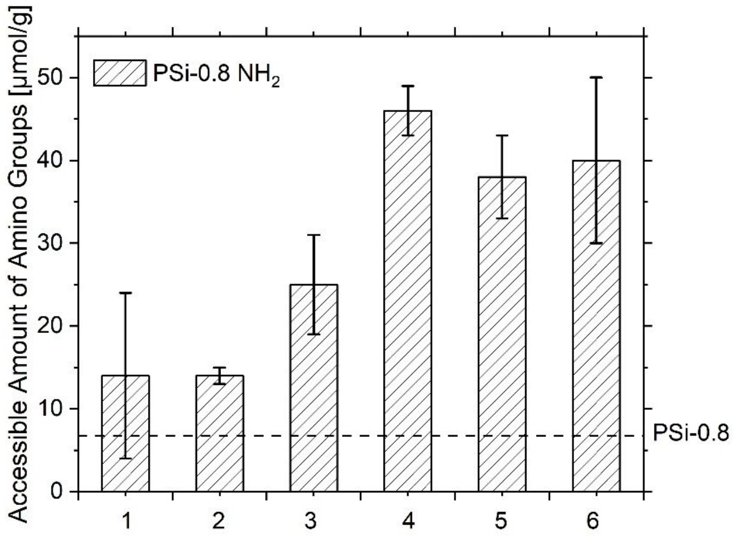

Figure S16. Results of the Fluram assay obtained for the differently modified PSi-0.8 NH_2; dashed line indicates the negative control.

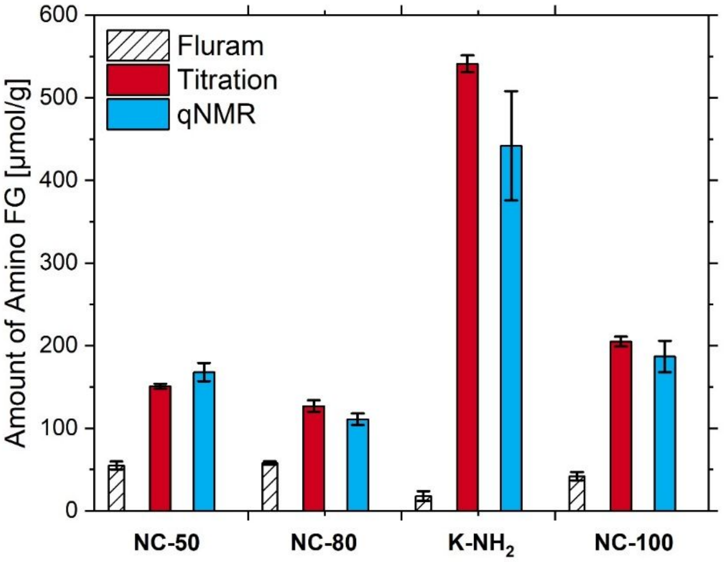

Figure S17. Comparison of potentiometric titration with the optical Fluram assay and qNMR measurements for different sized particles. The particle size of the NC samples does not have a significant influence on the difference results obtained by qNMR and titration, while the size distribution of the bimodal K-NH_2 (Klebosol-NH_2) show a significant influence. Additionally, the NC samples with nearly a monolayer of APTES show a smaller difference between the reporter-accessible amount and the total amount of amino FGs (factor around 2.7) than the bimodal K-NH_2 (Klebosol-NH_2) with multilayer structure (factor around 3.5).

Table S6. Overview of the comparison of the measured total amount and reporter-accessible amount of amino FGs for the various custom-made and commercial SiO_2 NPs using the potentiometric back titration and the Fluram assay.

Particle Name	Measured total amount of amino FGs [µmol/g]	Measured reporter accessible amount of amino FGs [µmol/g]	Reporter dependent factor
NC-50	151 ± 3	55 ± 5	2.75
NanoChOp-01	577 ± 4	72 ± 13	8.01
Sicastar 50	394 ± 6	186 ± 22	2.12
SiO_2-NH_2-50	505 ± 1	35 ± 6	14.43
NC-80	127 ± 7	58 ± 2	2.19
Sicastar 70	351 ± 5	99 ± 10	3.55
Klebosol-NH_2	541 ± 10	18 ± 6	30.06
NanoChOp-06	694 ± 1	62 ± 5	11.19
NC-100	205 ± 6	42 ± 5	4.56
Sicastar 100	474 ± 9	62 ± 14	7.65
PSi-0.1-NH_2	394 ± 9	64 ± 6	6.16

BAM SiO_2-100 NH_2 high	370 ± 24	62 ± 14	5.97
SiO_2-NH_2 100	375 ± 9	51 ± 7	7.35
micro450	363 ± 1	37 ± 3	9.81
NC-120	176 ± 12	83 ± 10	2.12
Sicastar 200	451 ± 5	46 ± 4	9.80

6. Automation

Although manual titration remains popular in laboratories and industries due to its perceived low cost and simplicity, accurate and reproducible results depend on factors such as transcription, maintenance/cleaning, sample preparation, and expirience of the operator. Automated titrators, on the other hand, are designed to simplify the titration process, making it more reliable and reproducible. They use highly precise motor-driven piston burette to dispense the titrant in extremely small increments (0.010 mL), that are difficult to realize for manual titration and are also usable in online measurements. Therefore we tested our potentiometric back titration approach with two commercially available automated titrators, a pH module 867 with two dosing units 800 Dosino and a pH micro glass electrode with ceramic diaphragm (6.0234.100, 5 kOhm) from Metrohm (Germany) and a technically more advanced Metrohm 815 Robotic USB Sample Processor XL with 10 mL 907 Titrando dosing units and a combined pH electrode with fixed ground-joint diaphragm (Ecotrode Plus, 5 kOhm). Both titrators were controlled by the tiamo software (Metrohm). The electrodes were stored, cleaned and activated according to the instructions of the manufacturer, while the titrators were calibrated prior the measurements. The temperature was held constant (23 ± 1 °C) during titrations. An addition of 0.02 mL per step was chosen. For the automated titration we optimized the concentration of the acid/base, the amount of utilized sample and the stop criterion to reduce the measurement time. The titration

With this simple automation, the hands-on preparation time for titer determination (green) and the titration step (Measurement & Evaluation, red) was significantly reduced from an average of 5 – 7 h to 2 – 3 h. In an 8-hour working day, one sample can be measured in triplicate manually as illustrated in Figure S19. Depending on the selected program and increment size,

automated titration requires only 0.75 – 1.5 hour per sample while maintaining high accuracy.With semi-automation, 2–3 samples can be processed per day, and with full automation, the assays can theoretically be performed continuously (24/7).

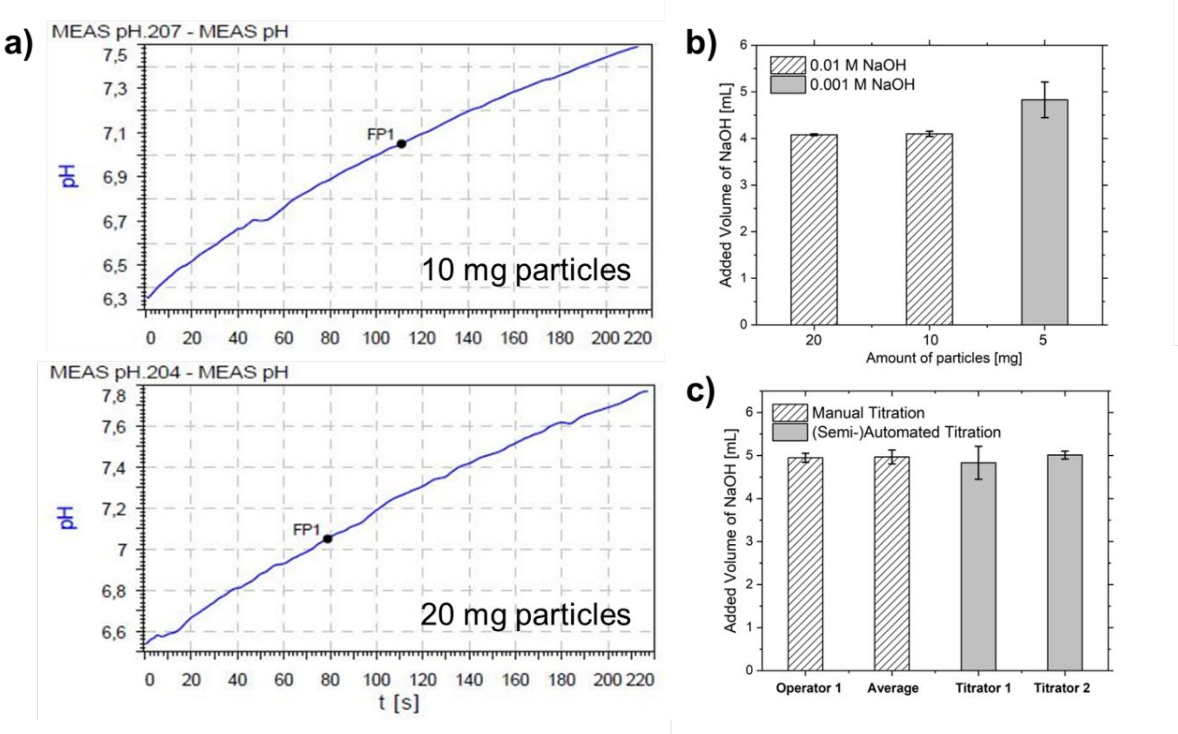

Figure S18. Comparison of (semi-)automated vs manual titration; a) Endpoints of the measurements for different concentrations with optimized stop criterion to reduce measurement time; b) comparison of the added volume of NaOH for different particle concentrations and base concentrations; c) comparison between the manually performed titration by a well-trained operator (operator 1), the average result of all operators and the automated titration by the two different titrators.

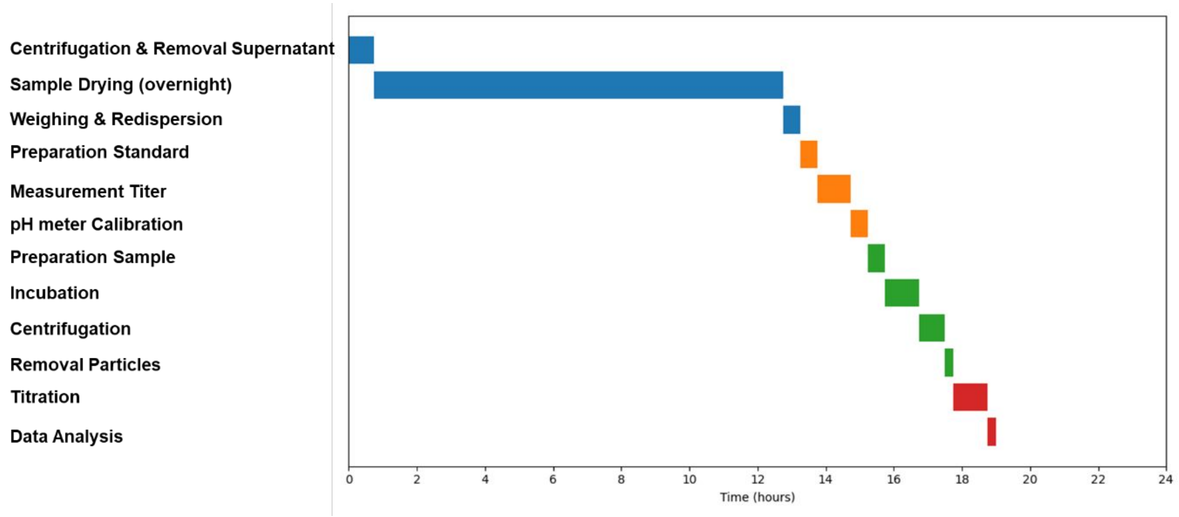

Figure S19. Gantt chart visualizing the estimated time of the titration workflow manually performed by a well-trained operator. Each bar represents a task and its estimated duration in hours (blue: Sample Preparation; orange: Titer Determination; green: Sample Incubation; red: Measurement & Evaluation).

7. Method adaptation for potentiometric back titration to other functional surface groups and types of nanoparticles

Finally, in a proof-of-concept approach, our potentiometric back titration method originally developed for aminated SiO_2 NPs was successfully adapted, to other FGs and particle types by tailoring the titration conditions to the specific acid-base properties of the surface functionalities. Aas representative examples, we chose custom-made aminated polystyrene NPs (PS-NH_2) prepared at BAM and commercial carboxylated SiO_2 NPs (PSI-0.1-COOH, Kisker Biotech GmbH & Co. KG, Germany). Prior to the titration, the pH stability of the polystyrene NPs was assessed. Therefore, a suspension of 10 mg/mL polystyrene NPs in MilliQ-water was prepared, and the initial pH was measured. The pH was then adjusted in 0.5 steps using HCl or NaOH, and the absorbance of each sample was recorded using a UV-Vis spectrophotometer (Varian Cary5000, Agilent, US). The evaluation focused thereby on the shape of the absorption curves, as exemplified in Figure S20 a), which correlates with particle size and hence particle integrity. Similar to the aminated SiO_2 NPs, the polystyrene NPs remained stable within the pH range of 3 to 10.

In addition, the carboxylated SiO_2 NPs required modifications in titrant selection to ensure an accurate endpoint detection. Due to the acidic nature of the surface functional groups, the method was reversed: the carboxylated particles were reacted with standardized NaOH, and the unreacted base in the supernatant was titrated with standardized HCl. The volume of HCl required to reach the equivalence point indicated the amount of unreacted NaOH. The results, shown in Figures S20 b) and S20 c), demonstrate the method's versatility and robustness, enabling quantitative analysis of surface FGs across a broad spectrum of modified nanomaterials.

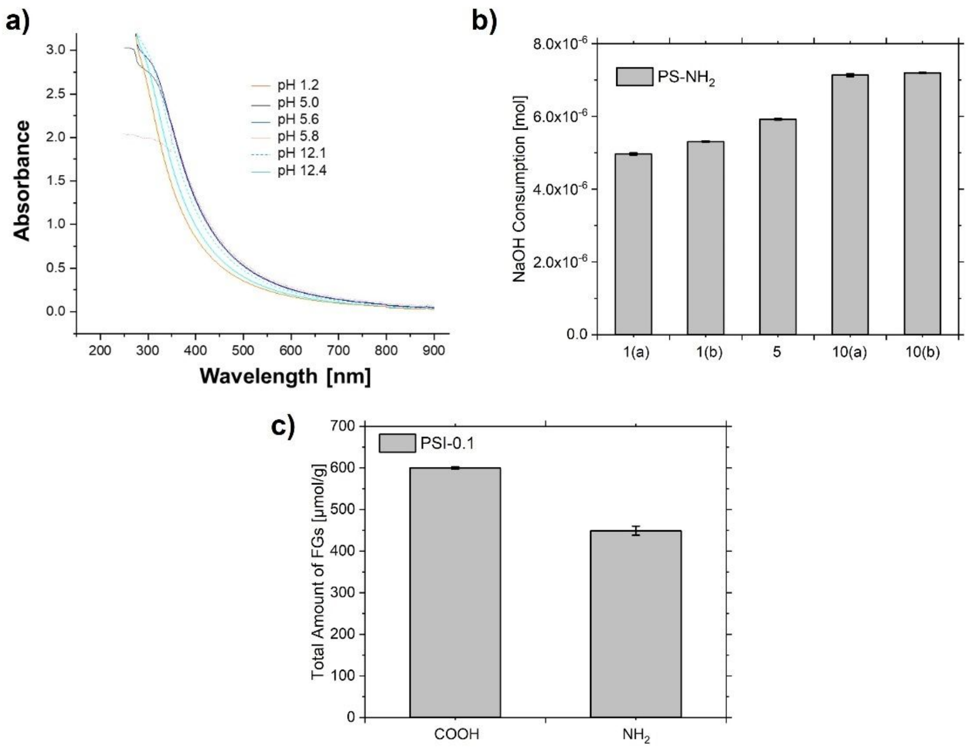

Figure S20. a) pH stability study of polystyrene NPs by measuring absorbance at different pH values. b) Screening of the potential influence of varying equivalents of amino ligand precursor used during polystyrene NP synthesis, assessed by measuring NaOH consumption via potentiometric back titration. Batch-to-batch variation is illustrated as batch (a) and batch (b). c) Results of the potentiometric back titration of commercial SiO_2 NPs (PSI-0.1) functionalized with NH_2 and COOH groups. The results were validated using TGA, showing mass losses of 4.47% for PSI-0.1 COOH and 3.99% for PSI-0.1 NH_2.

8. References

[1] Tavernaro, I.; Cavelius, C.; Peuschel, H.; Kraegeloh, A. Bright fluorescent silica-nanoparticle probes for high-resolution STED and confocal microscopy. *Beilstein Journal of Nanotechnology* 2017, *8*, 1283-1296.

[2] Tavernaro, I.; Matiushkina, A.; Rother, K. S.; Mating, C.; Resch-Genger, U. Exploring the potential of simple automation concepts for quantifying functional groups on nanomaterials with optical assays. *Nano Research* 2024, *17*, 10119-10126.

[3] Tourbin, M.; Frances, C. A Survey of Complementary Methods for the Characterization of Dense Colloidal Silica. Particle & Particle Systems Characterization 2007, 24, 411-423.

[4] International Organization for Standardization ISO 22412:2017, Particle size analysis - Dynamic light scattering (DLS) method; 2017.

[5] International Organization for Standardization ISO 19430:2016, Particle size analysis-Particle tracking analysis (PTA) method; 2016.

[6] ASTM E2834-12 Standard Guide for Measurement of Particle Size Distribution of Nanomaterials in Suspension by
Nanoparticle Tracking Analysis (NTA); 2012.

[7] Jung, H.-S.;Moon, D.-S.;Lee, J.-K.; Gu, H. C. Quantitative Analysis and Efficient Surface Modification of Silica Nanoparticles. Journal of Nanomaterials 2012, 2012

[8] Kotsyuda, S. S.;Tomina, V. V.;Zub, Y. L.;Furtat, I. M.;Lebed, A. P.;Vaclavikova, M.; Melnyk, I. V. Bifunctional silica nanospheres with 3-aminopropyl and phenyl groups. Synthesis approach and prospects of their applications. Applied Surface Science 2017, 420, 782-791.

[9] Fickert, J.; Rupper, P.;Graf, R.;Landfester, K.; Crespy, D. Design and characterization of functionalized silica nanocontainers for self-healing materials. J. Mater. Chem. 2012, 22, 2286-2291

[10] Szymanek, K.;Charmas, R.; Piasecki, W. Investigations of mechanism of Ca2+ adsorption on silica and alumina based on Ca-ISE monitoring, potentiometric titration, electrokinetic measurements and surface complexation modeling. Adsorption 2020, 27, 105-115

[11] Kunc, F.;Balhara, V.;Sun, Y.;Daroszewska, M.;Jakubek, Z. J.;Hill, M.;Brinkmann, A.; Johnston, L. J. Quantification of surface functional groups on silica nanoparticles: comparison of thermogravimetric analysis and quantitative NMR. Analyst 2019, 144, 5589-5599.

[12] Sun, Y.;Kunc, F.;Balhara, V.;Coleman, B.;Kodra, O.;Raza, M.;Chen, M.;Brinkmann, A.;Lopinski, G. P.; Johnston, L. J. Quantification of amine functional groups on silica nanoparticles: a multi-method approach. Nanoscale Adv 2019, 1, 1598-1607.

[13] Kunc, F.;Nirmalananthan-Budau, N.;Rühle, B.;Sun, Y.;Johnston, L. J.; Resch-Genger, U. Interlaboratory Comparison on the Quantification of Total and Accessible Amine Groups on Silica Nanoparticles with qNMR and Optical Assays. Analytical Chemistry 2021, 93, 15271-15278.
